# N^6^-methyladenosine modification of REG1α facilitates colorectal cancer progression via β-catenin/MYC/LDHA axis mediated glycolytic reprogramming

**DOI:** 10.1038/s41419-023-06067-6

**Published:** 2023-08-25

**Authors:** Mingxia Zhou, Jing He, Yingxia Li, Libin Jiang, Jiaxuan Ran, Chang Wang, Chenxi Ju, Dan Du, Xinyu Xu, Xuexin Wang, Hongle Li, Fucheng He, Hongtao Wen

**Affiliations:** 1 Department of Gastroenterology, The First Affiliated Hospital of Zhengzhou University, Zhengzhou, Henan China; 2 Department of Breast Surgery, The First Affiliated Hospital of Zhengzhou University, Zhengzhou, Henan China; 3 Department of Medical Laboratory, The First Affiliated Hospital of Zhengzhou University, Zhengzhou, Henan China; 4 Department of Molecular Pathology, The Affiliated Cancer Hospital of Zhengzhou University, Zhengzhou, Henan China

**Keywords:** Colorectal cancer, Tumour biomarkers

## Abstract

Aerobic glycolysis has been considered as a hallmark of colorectal cancer (CRC). However, the potential functional regulators of glycolysis in CRC remains to be elucidated. In the current study, we found that Regenerating islet-derived protein 1-alpha (REG1α) was significantly increased in both CRC tissues and serum, and positively associated with CRC patients’ lymph node metastasis, advanced tumor stage, and unfavorable prognosis. Ectopic expression of REG1α contributed to various tumorigenic properties, including cell proliferation, cell cycle, migration, invasion, and glycolysis. In contrast, REG1α deficiency in CRC cells attenuated malignant properties and glucose metabolism. Mechanically, REG1α promoted CRC proliferation and metastasis via β-catenin/MYC axis-mediated glycolysis upregulation. Moreover, the malignant behaviors governed by REG1α could be effectively abolished by silencing of Wnt/β-catenin/MYC axis or glycolysis process using specific inhibitors. Besides, REG1α expression was mediated by METTL3 in an m^6^A-dependent manner. Overall, our work defines a novel regulatory model of the METTL3/REG1α/β-catenin/MYC axis in CRC, which indicates that REG1α could function as a novel biomarker and a potential therapeutic target for patients with CRC.

## Introduction

According to a recent global cancer statistics report, colorectal cancer (CRC) is the third most common cancer and ranks second for cancer-related mortality globally [1, 2]. Clinical studies have shown that CRC prefers to metastasize to the liver and other organs, the 5-year survival rate of early CRC is around 90%, but this number declines to 15% in metastatic CRC. With the characteristics of rapid progression, unpleasant therapeutic responses, and adverse prognosis, CRC imposes considerable financial and public health burdens worldwide [3]. The development of CRC involves a series of complicated genetic, epigenetic and environmental changes. However, the underlying molecular mechanisms are still unclear so far. Therefore, it is imperative to explore effective biomarkers and novel therapeutic targets for the diagnosis and treatment of CRC.

Deregulating cellular metabolism is a universal hallmark of cancer initiation and progression [4]. Increasing studies have demonstrated that altered energy metabolism typified by enhanced aerobic glycolysis, increased lactate production as well as accelerated glucose consumption as the primary energy source even in the normoxic condition, which is also well-known as “Warburg effect”, is emerging as an essential hallmark in CRC tumor cells [5, 6]. Glycolysis, which yields only 2 ATP molecules in the conversion of glucose to lactate, although seems a senseless waste for tumor cells to choose glycolysis, the process contributes to tumor cells insensitive to hypoxic conditions, an abundant supplement of nucleosides and amino acids, and rapidly energy production [7, 8]. Moreover, lactate effectively facilitates tumor invasion by promoting migration, angiogenesis, radioresistance, and immune escape [9]. The redirection of glucose metabolism is characterized by overexpressed crucial effectors in the glycolytic pathway, including some enzymes responsible for the promotion of every single step in the glycolysis cascade, as well as some specific glucose membrane transporters [10]. Accumulating evidence suggests that glycolysis is driven by various oncogenic signaling pathways, including Wnt/β-catenin, PI3K/AKT, and MAPK signaling. However, the specific regulatory factors and underlying signaling pathway of glycolysis in CRC remain to be explored.

The human regenerating gene (REG) family are secreted proteins belonging to the C-type lectin superfamily [11, 12]. Reg-related genes containing REG 1α, REG 1β, REG III, REG IV, and HIP/PAP five members, and the first REG protein was named for its function in β-cell regeneration in rat regenerating pancreatic islets [13]. Although studies of this gene family are still spare, recent evidence indicated that REG-related genes might function as tissue mitogens [14, 15]. Previous study found that REG1α expression might be useful for early CRC diagnosis with a sensitivity of 90.6% and a specificity of 77.9% from DNA microarray analysis [16]. Another study by Wang et al. showed that inhibition of REG1α enhances the sensitivity to 5-Fluorouracil of CRC cells [17]. However, the precise molecular mechanisms underlying REG1α in CRC initiation and progression remain elusive. Herein, we elaborated the first comprehensive study concerning the biological function, clinical implication, and molecular mechanisms of REG1α in CRC. We discovered that the aberrantly expressed REG1α promoted CRC cells proliferation, prevented apoptosis, expedited cell cycle, migration and glucose metabolism in vitro. Mechanically, REG1α regulated tumorigenic properties via β-catenin/MYC/LDHA axis-mediated glycolysis and was modified by m^6^A methyltransferase METTL3. Taken together, our findings underscore the potential role of REG1α as a novel candidate biomarker and therapeutic target for CRC progression.

## Results

### High REG1α expression predicts poor prognosis of CRC patients

**Fig. 1:**
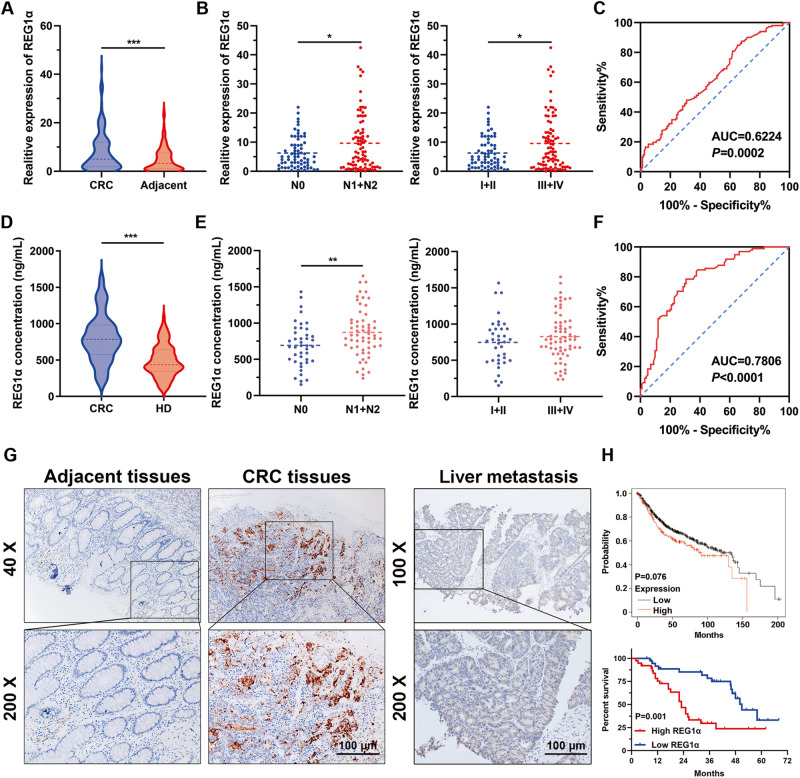
Upregulation of REG1α in CRC tissues is correlated with poor prognosis of CRC patients. **A** qRT-PCR analysis of REG1α mRNA level in primary CRC samples and matched adjacent normal colorectal tissues (*n* = 152). **B** Statistical analysis REG1α expression levels in CRC patients with lymph node metastasis (*n* = 68 vs. *n* = 84) and different TNM stages (*n* = 66 vs. *n* = 86). **C** ROC curve analyses for REG1α in CRC samples and adjacent normal colorectal tissues. **D** Serum REG1α level in CRC patients (*n* = 101) and healthy controls (*n* = 98) were analyzed using Human REG1α ELISA Kit. **E** The correlation between serum REG1α level and lymph node status (*n* = 42 vs. *n* = 59) and pathological stage (*n* = 37 vs. *n* = 64) in CRC patients. **F** ROC curve analyses for serum REG1α in CRC patients and healthy donors. **G** Immunohistochemistry staining showing the expression of REG1α in CRC tissues relative to paracancerous tissues and liver metastasis foci. Magnification: ×200, Scale bar = 100 μm. **H** Kaplan–Meier analysis revealed that CRC patients with high REG1α expression had worse overall survival rate than those with low REG1α expression. Upper panel: Kaplan-Meier Plotter (*n* = 551); lower panel: ZZU cohort (*n* = 76). Significant differences were shown by *P < 0.05, **P < 0.01 and ***P < 0.001.

To explore the key molecules that potentially participating in the progression of CRC, we analyzed differential gene expression using the public GEO databases. A total of 374 CRC tissues and 243 adjacent normal tissues from GSE18105, GSE20916, GSE28000 and GSE44076 confirmed the significant elevated REG1α in human CRC tissues compared to adjacent normal colon tissues (Fig. S1A). Meanwhile, the expression profiles from TCGA datasets also pinpointed that REG1α in colon adenocarcinoma was considerably increased when compared with normal tissues (Fig. S1B). We further evaluated REG1α expression in cohort 1, containing 152 pair of matched CRC and adjacent normal tissues by qRT-PCR. As shown in Fig. 1A, REG1α mRNA expression was drastically upregulated in CRC tissues in comparison with their matched adjacent normal counterparts. We next determined the correlation between REG1α expression and clinicopathological characteristics in 152 CRC patients. Of note, high REG1α expression showed no association with age, gender, tumor size, grade, and invasion, but was closely linked to lymph node metastasis and advanced TNM stage of CRC patients (Fig. 1B and Supplementary Table S1). ROC curves and area under the ROC curves (AUC) were then analyzed to assess the diagnostic capacity of REG1α in CRC samples and adjacent normal counterparts. The results indicated that the AUC for REG1α was 0.6224 (95% CI: 0.5601–0.6848, *P* = 0.0002, Fig. 1C). We also observed higher REG1α protein level in the blood samples of 101 CRC patients compared with 98 healthy donors (cohort 2) using commercial ELISA kit, which were consistent with the aforementioned qRT-PCR results (Fig. 1D). CRC patients with lymph node metastasis showed higher expression of REG1α in serum (Fig. 1E). Moreover, the serum REG1α level could well differentiate CRC patients from healthy donors, with an AUC of 0.7806 (95% CI: 0.7163– 0.8448, *P* < 0.0001, Fig. 1F). Immunohistochemistry (IHC) staining results and scores from 68 pairs of CRC and adjacent normal tissues (cohort 3) further corroborated these findings (Fig. 1G and S1C). Importantly, REG1α was also highly expressed in liver metastatic foci of CRC patients (Fig. 1G). Furthermore, we also noticed that CRC patients with high REG1α expression exhibited poor overall survival, as analyzed by Kaplan-Meier Plotter database and clinical information in our ZZU cohort (Fig. 1H). Taken together, our data clarified that REG1α is an appropriate diagnostic biomarker and prognostic indicator for CRC patients.

### REG1α promotes the malignant phenotypes of CRC cells in vitro

**Fig. 2:**
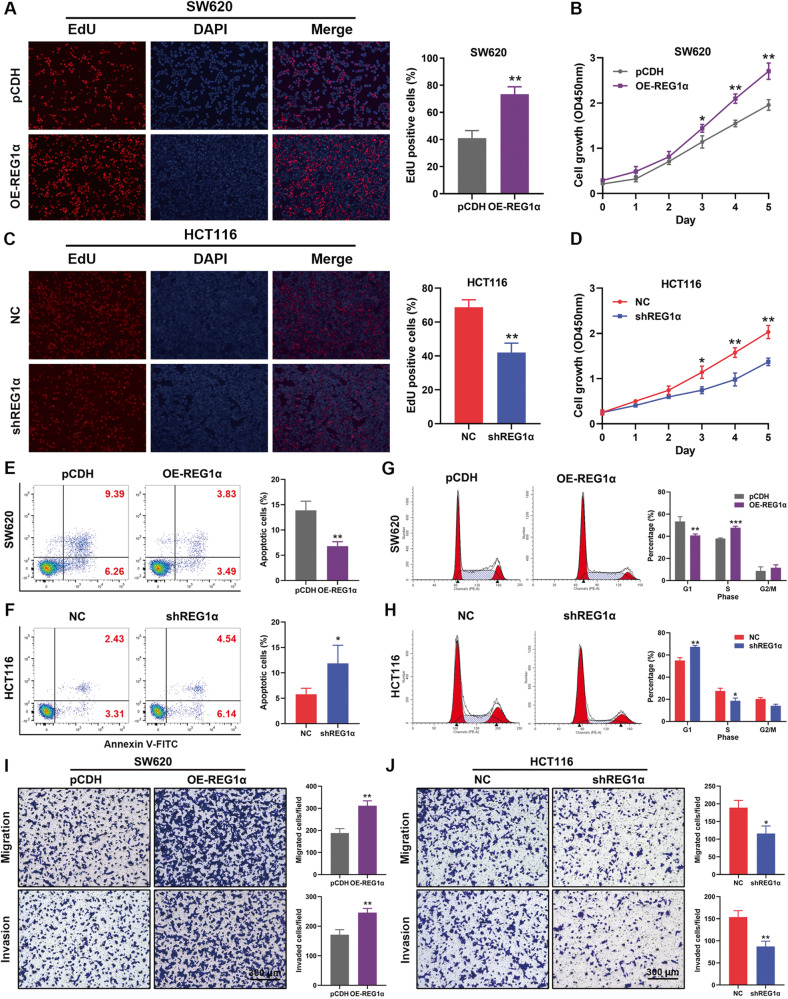
REG1α facilitates the malignant phenotypes of CRC cells in vitro. **A** Effects of REG1α overexpression on cell proliferation was detected by EdU incorporation assays. **B** Growth curve of SW620 cells with REG1α overexpression was determined by performing the CCK-8 assay for 5 days. **C** REG1α knockdown inhibited cell growth in HCT116 cells by EdU staining. **D** CCK-8 assay was used to assess cell viability in HCT116 cells with stable REG1α knockdown. **E**, **F** CRC cell apoptosis was determined by Annexin V-FITC/PI staining. **G**, **H** Flow cytometry was applied to compare the G1, S, G2/M phase of cell cycle in SW620 and HCT116 cells. **I**, **J** Representative images of migration (upper) and invasion (lower) experiments in SW620 (left) and HCT116 cells (right) and quantification of the relative migrated and invaded cell number. Scale bar = 100 μm. Data represent the mean ± SD (*P < 0.05, **P < 0.01, and ***P < 0.001).

We then compared the expression of REG1α in a series of CRC cell lines by qRT-PCR and western blot analysis. Notably, REG1α was heterogeneously upregulated in a panel of CRC cells, with HCT116 had the highest level, while SW620 cells showed the lowest level, whereas REG1α was silenced in normal colonic epithelial cells NCM460 and CCD841 (Fig. S2A). To delineate the potential role of REG1α in CRC progression, we stably knocked down the REG1α expression in HCT116 cells with specific shRNAs, and overexpressed REG1α expression in SW620 cells with low REG1α endogenous level. The expression status of REG1α in these cells were confirmed by qRT-PCR (Fig. S2B and C). EdU and CCK-8 assay was conducted to find out the effects of REG1α on cell proliferation. EdU immunofluorescence staining showed that overexpression of REG1α considerably accelerated the growth of SW620 cells, whereas knockdown of REG1α markedly inhibited the cell growth of HCT116 cells (Fig. 2A, C). CCK-8 assay also displayed the tumor-promoting impacts of REG1α on the viability of CRC cells (Fig. 2B, D). Besides, the results from colony-forming assays showed that forced expression of REG1α promoted the clonogenicity in SW620 cells. In contrast, knockdown of REG1α apparently restrained the clonogenicity in HCT116 cells (Fig. S2D and E). Next, we analyzed the cellular apoptosis and cell cycle distribution by flow cytometry after modulating REG1α expression in SW620 and HCT116 cells. We found that the apoptosis rate was exacerbated in shREG1α cells when compared to negative control (NC) cells. However, the apoptosis rate was potently declined in OE-REG1α cells (Fig. 2E, F). Furthermore, the cell cycle analysis showed that REG1α knockdown in HCT116 cells resulted in cell cycle arrest at the G1 phase. Conversely, overexpression of REG1α in SW620 cells markedly sped up the G1/S phase transition and augmented the S-phase percentage (Fig. 2G, H). These data suggested that REG1α accelerated CRC cell growth via regulating cellular apoptosis and cell cycle progression. In addition, Transwell assays with or without Matrigel were performed to assess the effects of REG1α on CRC cell mobility. As shown in Fig. 2I, ectopic REG1α obviously induced SW620 cell migration and invasion in comparison with the control cells. However, REG1α-depletion in HCT116 cells notably impaired the migration abilities (Fig. 2J). Taken together, these data disclosed the biological role of elevated REG1α expression in influencing the malignant characteristics of CRC cells.

### REG1α influences glucose metabolism in CRC cells

**Fig. 3:**
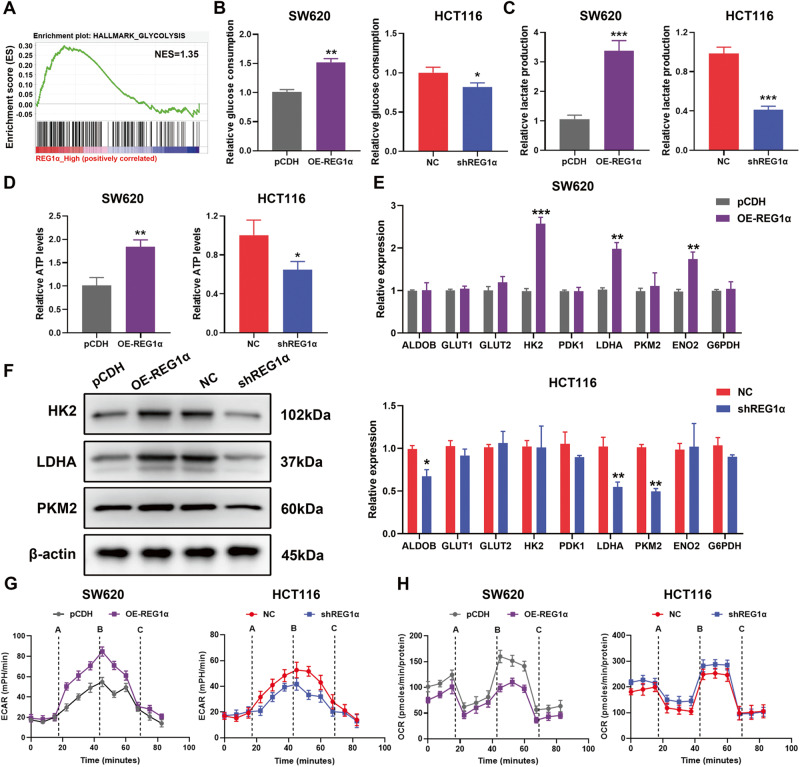
REG1α modulates glycolysis of CRC cells. **A** GSEA analysis of the TCGA colon adenocarcinoma datasets showed that glycolysis pathway was strongly associated with REG1α expression. **B** Forced expression of REG1α increased the uptake of glucose while knockdown of REG1α had an opposite effect. **C** Relative lactate production in CRC cells with REG1α overexpression or knockdown was determined by lactate assay. **D** ATP production was examined in SW620 and HCT116 cells with indicated vectors. **E** The effects of REG1α on the mRNA levels of glycolytic enzymes and glucose transporters in SW620 (upper) and HCT116 (lower) cells were detected by qRT-PCR. **F** The effects of REG1α on the protein levels of several glycolysis enzymes were analyzed by western blot. **G** After indicated treatment on CRC cells, the extracellular acidification rate (ECAR) was measured (A: Glucose B: Oligomycin C: 2-DG). **H** Oxygen consumption rate (OCR) was determined in CRC cells transfected with certain vectors and exposed to Oligomycin, Trifluoromethoxy carbonylcyanide phenylhydrazone (FCCP) and Rotenone. Data were expressed as the mean ± SD from three independent experiments. Significant differences were shown by *P < 0.05, **P < 0.01, and ***P < 0.001.

To better understand the possible mechanisms that REG1α participated in CRC tumorigenesis, we undertook Gene Set Enrichment Analysis (GSEA) using TCGA datasets of CRC patients. Interestingly, GSEA results demonstrated that glycolysis is among the most significantly altered hallmarks in CRC patients with high REG1α expression (Fig. 3A). Growing evidence highlighted the crucial role of aerobic glycolysis in maintaining the malignant features of tumor cells, such as proliferation and invasion. Therefore, we explored the effects of REG1α on CRC cell glucose metabolism. As shown in Fig. 3B, forced expression of REG1α in SW620 cells elevated glucose consumption while silencing REG1α in HCT116 cells exhibited an opposite effect. In parallel, overexpression of REG1α led to the elevated lactate and ATP generation, whereas knockdown of REG1α inhibited the cellular lactate and ATP production (Fig. 3C, D). Moreover, the additional treatment of glycolysis inhibitor 2-DG in REG1α overexpressed cells greatly reduced glycolysis, cell growth, colony formation, migration, and invasion in a concentration-dependent manner (Fig. S3A-F). We further evaluated the expression of major glycolytic enzymes and glucose transporters to figure out the molecular mediators sustaining the glycolytic rate. Overexpression of REG1α boosted the transcriptional levels of multiple glycolytic enzymes and related encoding genes, including HK2, LDHA, and ENO2, whereas knockdown of REG1α decreased ALDOB, LDHA, and PKM2 expression, as evidenced by qRT-PCR assays (Fig. 3E). As shown in Fig. 3F, REG1α overexpression significantly enhanced the protein levels of HK2, LDHA, and PKM2, whereas REG1α knockdown did the opposite. In addition, REG1α-overexpressed SW620 cells showed higher extracellular acidification rate (ECAR) and lower oxygen consumption rates (OCR) levels compared with the control cells, whereas ablation of REG1α in HCT116 cells prominently weakened ECAR and increased OCR levels (Fig. 3G, H). Collectively, these findings indicated that REG1α evidently exaggerated glycolysis in CRC cells.

### MYC activates the transcription of LDHA and participates in REG1α-stimulated glycolysis in CRC cells

**Fig. 4:**
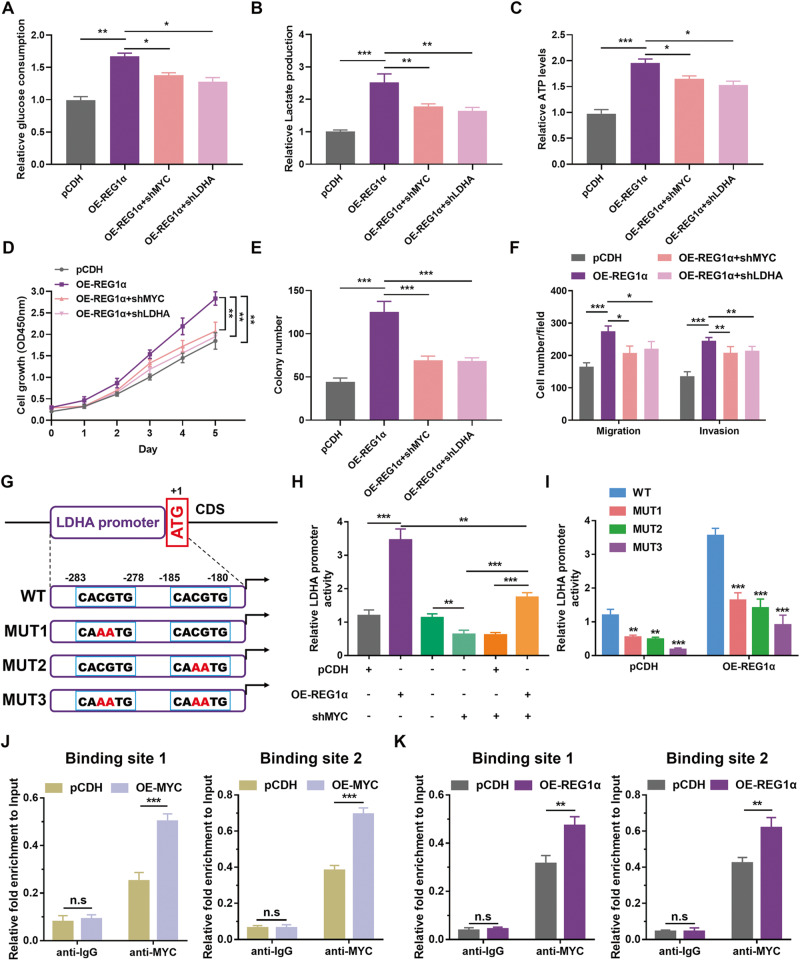
MYC and LDHA are the functional target genes of REG1α in CRC. **A**–**C** Relative fold changes of glucose consumption (**A**), lactate concentration (**B**), ATP production (**C**) in REG1α-overexpressed SW620 cells transfected with shMYC or shLDHA vectors compared to corresponding control cells. **D**, **E** Cell growth were determined in REG1α-overexpressed SW620 cells with or without further inhibition of MYC and LDHA employing CCK-8 assays (**D**) and colony-formation assays (**E**). **F** Transwell assay was performed in control and REG1α overexpression cells with either depletion of MYC or LDHA. **G** Schematic illustration showing the predicted binding sites of MYC at the human LDHA promoter region. Mutant vectors were constructed by replacing CACGTG motifs with CAAATG alone or in combinations. **H** Relative luciferase activities of the LDHA promoter in SW620 cells stably expressing REG1α with or without further depletion of MYC were examined. **I** Luciferase activities of different mutant LDHA promoter reporters in control and REG1α-overexpressed cells were quantified and normalized to wild type. **J** ChIP assay analysis of the enrichment of MYC on the separated binding sites of the promoter of LDHA in SW620 cells. **K** Elevated expression of REG1α increased the enrichment of MYC on LDHA promoter. Data were represented as the mean ± SD from three independent experiments. Significant differences were shown by *P < 0.05, **P < 0.01, ***P < 0.001 and n.s., not significant.

Previous studies have extrapolated an exemplary role of MYC in the metabolic reprogramming of tumor cells [18]. Therefore, we investigated whether MYC participated in the REG1α-mediated increment of aerobic glycolysis. Notably, GSEA enrichment analysis demonstrated that MYC and Cyclin D1 were prominently upregulated in CRC patients with high REG1α expression (Fig. S4A). Three GEO datasets GSE20916, GSE44076, and GSE18105 all exhibited a pronounced increase of MYC expression in CRC tissues compared to noncancerous tissues (Fig. S4B). As expected, we found MYC expression was upregulated in 62 pairs of matched CRC and normal tissues by qRT-PCR, which was in accordance with the above data-mining results (Fig. S4C and S4D). We previously found that LDHA exhibited the most noticeable change in mRNA and protein expression level upon disturbance or overexpression of REG1α. To determine whether MYC or LDHA participated in REG1α mediated glycolysis, we established SW620 cells stably expressing an shRNA targeting MYC or LDHA. Intriguingly, inhibiting MYC and LDHA remarkably suppressed the increased glucose consumption, cellular lactate, and ATP generation produced by REG1α (Fig. 4A–C). Meanwhile, inhibition of MYC or LDHA abolished the enhanced cell proliferation, colony-forming, migration, and invasion capacities by REG1α (Fig. 4D–F). These results unveiled that both MYC and LDHA are oncogenic modulators in REG1α-induced glycolysis as well as cell growth and metastasis. Besides, overexpressing MYC in SW620 cells noticeably promoted glycolysis and malignant behaviors, including cell growth and metastasis, while inhibiting LDHA in MYC-expressing cells effectively reversed the facilitation (Fig. S4E–J). To further elucidate the interaction of MYC and LDHA, we searched ~2000 bp of the promoter regions of LDHA genes for putative MYC binding sites, and constructed the promoter reporters, including the predicted binding sites or mutant vectors (replacing CACGTG motifs with CAAATG alone or in combinations) through JASPAR database (https://jaspar.genereg.net, Fig. 4G). The results indicated that forced expression of REG1α remarkably enhanced the LDHA promoter luciferase activities in SW620 cells, while depletion of MYC in REG1α-overexpression cells restrained this increment (Fig. 4H). Specifically, both mutation of site 1 and 2 lost REG1α-mediated activation of LDHA luciferase activity (Fig. 4I). Next, to examine whether MYC promotes LDHA expression by directly binding to its promoter region, chromatin immunoprecipitation (ChIP) assay was adopted, and the results showed that the two predetermined sites sequences were dramatically precipitated by MYC antibody (Fig. 4J). Meanwhile, the elevated expression of REG1α in SW620 cells also augmented the enrichment of MYC on LDHA promoter (Fig. 4K). Collectively, these findings elucidated that MYC and LDHA are responsible for REG1α-induced glycolysis, and MYC activates the transcription of LDHA in CRC cells.

### REG1α-induced MYC upregulation is mediated by the activation of Wnt/β-catenin signaling

**Fig. 5:**
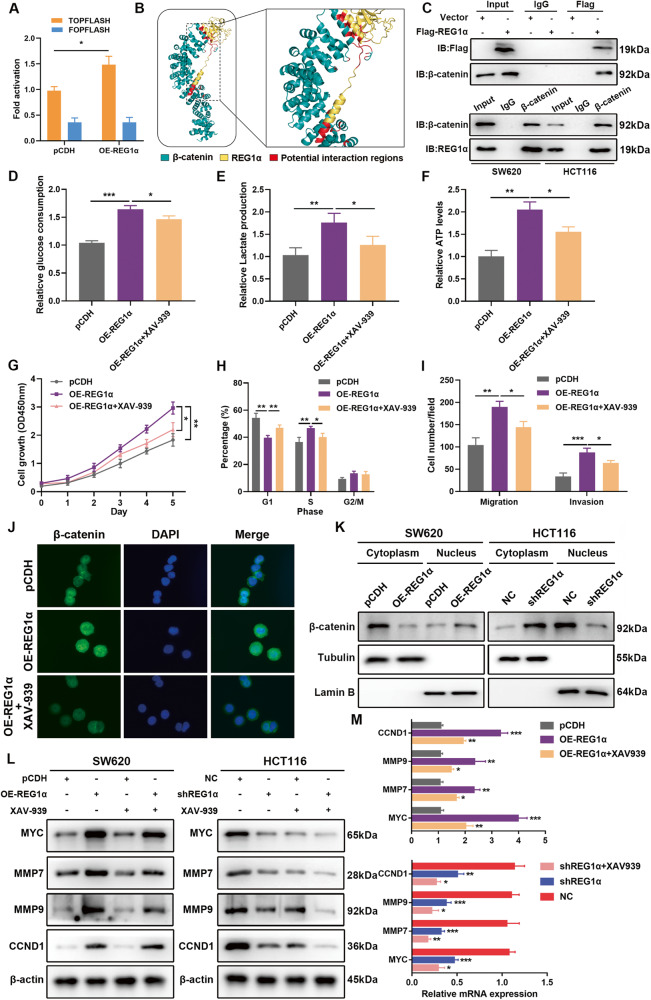
REG1α physically interacts with β-catenin and promotes the nuclear translocation of β-catenin in CRC cells. **A** TOP/FOPFLASH luciferase reporter assays were performed to measure the effects of REG1α on Wnt/β-catenin activity. The FOPFLASH exhibited no remarkable reporter activity. **B** Interactions between REG1α and β-catenin were modeled by molecular docking studies. **C** Validation of the interaction between REG1α and β-catenin in CRC cells by co-immunoprecipitation using anti-Flag or anti-β-catenin antibody, with IgG as negative control. **D**–**F** Quantification of in glucose consumption (**D**), lactate concentration (**E**), ATP production (**F**) in control and REG1α-overexpressed SW620 cells treated with DMSO or β-catenin inhibitor XAV-939 for 48 h. **G**–**I** The effects of β-catenin inhibitor XAV-939 on the REG1α-mediated malignant phenotypes in SW620 cells, including proliferation (**G**), cell cycle (**H**), migration and invasion (**I**), were detected by CCK-8, flow cytometry and Transwell assays. **J** Cellular location of β-catenin in SW620 cells was monitored by immunoflouresence assay. **K** Western blot analysis for the distribution of β-catenin in CRC cells upon overexpression or inhibiton of REG1α. Tubulin was loaded as a cytoplasmic marker and Lamin B was used as a nuclear marker. **L**, **M** Western blot (**L**) and qRT-PCR (**M**) uncovered the change of protein or mRNA level of MYC, MMP7, MMP9 and CCND1 after treatment with XAV-939 in REG1α-overexpressed or REG1α-silenced CRC cells. Significant differences were shown by *P < 0.05, **P < 0.01, and ***P < 0.001.

We next aim to figure out the exact mechanism of MYC upregulation by REG1α. Literature reported that MYC transcription could be activated by a series of signaling pathways, including JAK/STAT, MAPK, TGF-β and Wnt/β-catenin axis [19]. Therefore, we treated CRC cells with the STAT3 pathway inhibitor Stattic, ERK/MAPK pathway inhibitor PD98059, TGF-β1 receptor antagonist SB431542, Wnt/β-catenin pathway inhibitor XAV-939 to measure the change of expression of MYC. Of note, only treating REG1α-overexpressing SW620 cells with XAV-939, the expression of MYC was remarkably down-regulated (Fig. S5A). Meanwhile, GSEA enrichment analysis showed that the Wnt signaling pathway was prominently positively associated with REG1α in CRC patients (Fig. S5B). To analyze the effects of REG1α on the activity of Wnt/β-catenin signaling in CRC cells, TOP/FOPFLASH reporter assay was conducted, and the results showed that overexpression of REG1α markedly augmented the transcriptional activity of β-catenin, whereas silencing REG1α impaired Wnt/β-catenin pathway activity (Fig. 5A). Then we carried out the molecular docking to evaluate the binding potential of β-catenin and REG1α. According to in-silico protein docking analysis, REG1α had a high affinity with β-catenin (Fig. 5B). To directly test the interaction between REG1α and β-catenin, we performed co-immunoprecipitation (co-IP) assays by using anti-Flag or anti-β-catenin antibodies, respectively, and found that REG1α could interact with β-catenin both exogenously and endogenously (Fig. 5C). We further utilized Wnt/β-catenin pathway-specific inhibitors XAV-939 to confirm the involvement of the Wnt/β-catenin axis on REG1α-stimulated glycolysis and malignant tumor phenotypes in CRC. As shown in Fig. 5D–I, XAV-939 alleviated the exacerbation of various malignant phenotypes mediated by exogenous REG1α in SW620 cells, including glucose consumption, cellular lactate generation, ATP production, cell proliferation, cell cycle, migration, and invasion. In addition, REG1α substantially accelerated the accumulation of β-catenin in the cell nucleus while decreased its level in cytoplasm, and XAV-939 administration ameliorated the translocation of β-catenin from the cytoplasm to nucleus (Fig. 5J, K). Besides, we detected the protein levels of MYC, Cyclin D1 (CCND1), MMP7 and MMP9 in REG1α-overexpressing SW620 and REG1α-suppressive HCT116 cells after the treatment with XAV-939. We noticed that REG1α significantly promoted the expression levels of β-catenin target proteins MYC, CCND1, MMP7, and MMP9, and XAV-939 administration effectively reversed this change (Fig. 5L, M). Taken together, these results suggested REG1α promoted MYC expression through activation of Wnt/β-catenin signaling in CRC cells.

### METTL3 regulates REG1α mRNA levels and stability in CRC

**Fig. 6:**
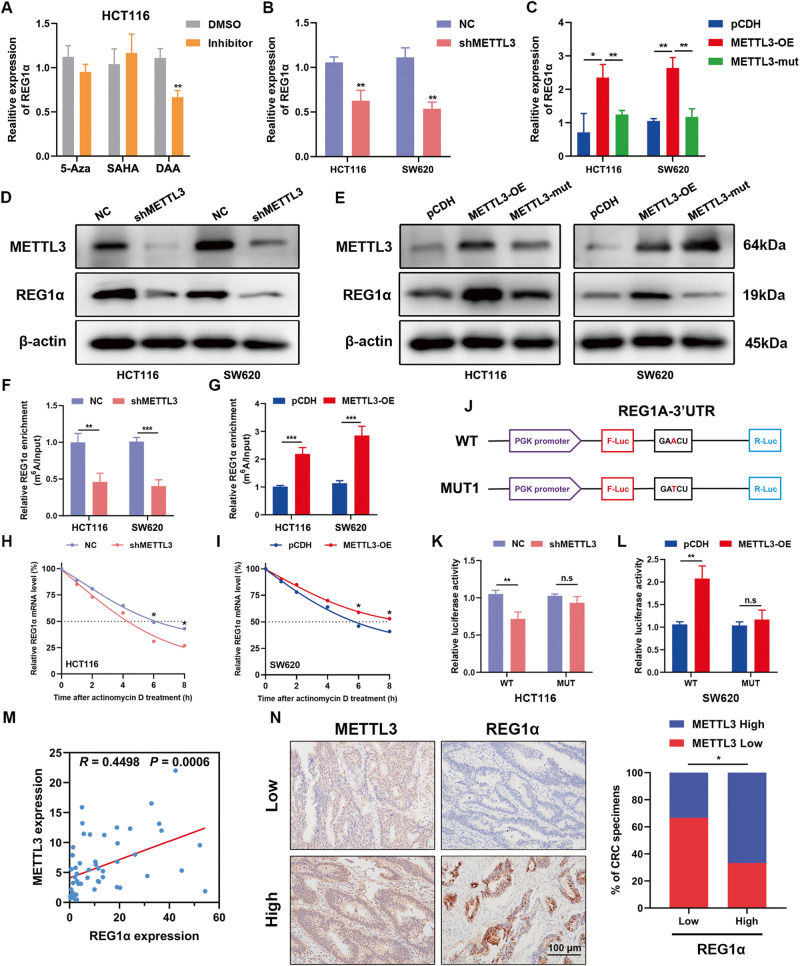
METTL3-mediated m^6^A modification of REG1α is responsible for its upregulation in CRC cells. **A** The mRNA levels of REG1α was measured by qRT-PCR after treatment with 5-Azacytidine (10 µM), SAHA (10 µM) or 3-Deazaadenosine (10 µM) for 24 h. **B**, **C** qRT-PCR detection of REG1α upon inhibition and overexpression wild type or catalytic mutant METTL3 in HCT116 and SW620 cells. **D**, **E** Western blot analyses for the expression of REG1α in METTL3-deficient or wild type and catalytic mutant METTL3 overexpression CRC cells. **F**, **G** Gene-specific m^6^A-qPCR validation of m^6^A levels on REG1α mRNA after inhibition or overexpression of METTL3 in HCT116 and SW620 cells. **H**, **I** The decay rate of REG1α mRNA in control and METTL3-silenced or METTL3-overexpressing CRC cells treated with actinomycin D (5 mg/mL) at the indicated time point. **J** Wild-type luciferase reporter embodied the REG1α 3’UTR with intact m^6^A sites, while mutant reporter was generated by replacing adenosine with thymine on m^6^A consensus motifs. **K**, **L** Relative activity of the WT or MUT luciferase reporters in METTL3-silenced or METTL3-overexpressed CRC cells was determined and normalized to Renilla activity. **M** The correlation between METTL3 and REG1α mRNA level was investigated by RT-PCR in a cohort of 55 primary CRC samples. **N** Representative immunohistochemical images of METTL3 and REG1α in CRC tissues (left). Correlation between METTL3 expression and REG1α IHC scores in another cohort of 48 CRC samples (right). Scale bar = 100 μm. Significant differences were shown by *P < 0.05, **P < 0.01, and ***P < 0.001.

Recent discoveries in N^6^-methyladenosine (m^6^A) modification have shed light on a novel layer of epitranscriptomics [20]. In order to ascertain why REG1α was highly expressed in colorectal cancer, we exposed HCT116 cells to DNA methyltransferase inhibitor 5-Aza-2’-deoxycytidine (5-Aza), pan histone deacetylase inhibitor Vorinostat (SAHA) or global methylation inhibitor 3-deazaadenosine (DAA) to evaluate the change of REG1α mRNA levels. The results showed that only DAA impaired the expression of REG1α (Fig. 6A). As previous studies have reported that m^6^A methyltransferase METTL3 also participated in the glucose metabolism of CRC [21]. Thus, we examined the impact of METTL3 level on REG1α expression, inhibition of METTL3 strongly decreased the mRNA and protein level of REG1α, while ectopic expression of METTL3 could upregulate but catalytic mutant METTL3 (MTase domain deletion) failed to increase the level of REG1α (Fig. 6B–E). According to the MeRIP-PCR results, the m^6^A level of REG1α was obviously decreased in METTL3-silenced CRC cells (Fig. 6F). In contrast, ectopic expression of METTL3 rendered the elevated m^6^A level of REG1α (Fig. 6G). Next, we found that METTL3 knockdown accelerated the decay of REG1α and forced expression of METTL3 could prolong the lifetime of the REG1α mRNAs (Fig. 6H–I). Predictive results from the online N^6^-methyladenosine modification site SRAMP (http://www.cuilab.cn/sramp) revealed that one m^6^A site existed in the REG1α transcript. By constructing wild-type or mutant m^6^A sites of REG1α luciferase reporter, we noticed that mutant of m^6^A modification sites of REG1α impaired METTL3-mediated activation of REG1α luciferase activity (Fig. 6J–L). To further support the vital role of REG1α for METTL3-stimulated glycolysis, we pre-knocked down REG1α in HCT116 cells following overexpression of METTL3, and examined glycolysis-related phenotypes. Overexpression of METTL3 drove glycolytic metabolism in colorectal cancer through increasing glucose uptake, lactic acid production and ATP level. However, deletion of REG1α obviously blocked METTL3-induced glycolysis (Fig. S6A–C). In addition, we found that cell proliferation, colony-formation and mobility abilities were partially weakened in a REG1α-dependent manner (Fig. S6D–F). At last, we analyzed the correlation between METTL3 and REG1α expression in CRC specimens using qRT-PCR and immunohistochemistry, the results indicated that REG1α was positively correlated with METTL3 level in CRC tissues (Fig. 6M–N). Overall, the aboving results demonstrated that METTL3 maintained the mRNA stability of REG1α, and REG1α is a functionally indispensable target of METTL3 in CRC.

### REG1α drives CRC cell growth and metastasis in an MYC-dependent manner in vivo

**Fig. 7:**
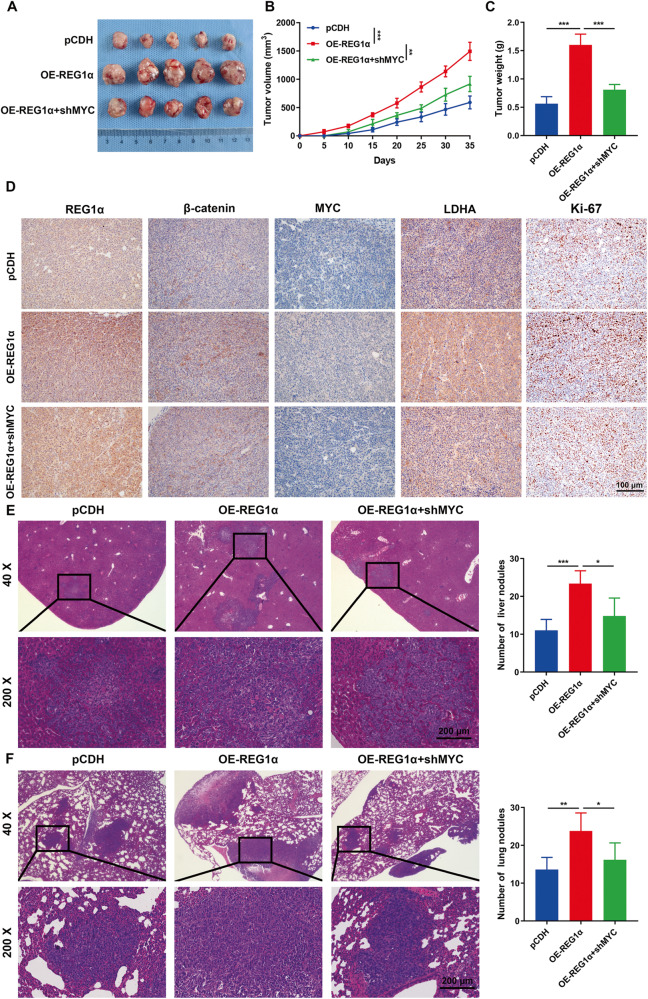
REG1α facilitates tumorigenesis and metastasis of CRC cells in a MYC-dependent manner. **A** Images of tumors formed in nude mice bearing control SW620 cells and REG1α-overexpression cells with or without MYC inhibition. **B** Tumor volumes were monitored every five days and tumor growth curves were shown. **C** The weight of xenograft tumors between different groups were measured at the endpoint of experiment. **D** Representative IHC images of REG1α, β-catenin, MYC, LDHA and Ki-67 staining in serial sections of tumor tissues isolated from subcutaneous models. **E** Representative images of H&E staining of liver sections in nude mice (*n* = 5 per group) after intrasplenic injection of SW620 cells. Statistical analysis of metastatic nodules was evaluated. **F** SW620 cells with indicated plasmids were injected into the tail veins of nude mice and the numbers of metastatic foci in the lung were quantified. Scale bar = 200 μm. Data were expressed as the mean ± SD. Significant differences were shown by *P < 0.05, **P < 0.01, and ***P < 0.001.

For exploration of the biological function of REG1α in vivo, we next selected immunodeficient mouse models as the research subject. REG1α-overexpressing SW620 cells with or without MYC inhibition, REG1α knockdown HCT116 cells transfected with or without OE-MYC plasmids and their corresponding control cells were injected subcutaneously into the armpits of nude mice in different groups, respectively. After five weeks of observation, tumors were harvested from mice. Compared with the control group, the mice implanted with OE-REG1α cells showed faster tumor formation and a remarkable increment of tumor growth and weight, whereas cells with MYC deficiency counteracted these effects (Fig. 7A–C). On the contrary, silencing of REG1α retarded the tumor growth in vivo and decreased the tumor burden, while co-transfection with OE-MYC reversed the changes (Fig. S7A–C). Moreover, immunohistochemistry stains further confirmed that growth-promoting tumors generated from REG1α-overexpressed CRC cells exhibited higher expression of β-catenin, MYC, LDHA and Ki-67 (Fig. 7D). In addition, the experimental liver and lung metastasis models were also generated to evaluate the effects of REG1α and MYC on tumor cell metastasis. We found that SW620 cells with stably overexpressing REG1α significantly increased the numbers of metastatic nodules in the liver and lung tissues of mice in comparison with the control group, as evidenced by histologic examination, while co-transfection with shMYC attenuated these changes (Fig. 7E, F). Inhibiting of REG1α suppressed the growth of metastatic nodules in the liver and lung tissues of mice, while the addition of OE-MYC transfection partially reversed the decline (Fig. S7D–E). Together, these in vivo data, corroborating the above in vitro results, concluded the oncogenic role of REG1α during the progression of CRC.

### Clinical correlation between REG1α, β-catenin, MYC, and LDHA in CRC

**Fig. 8:**
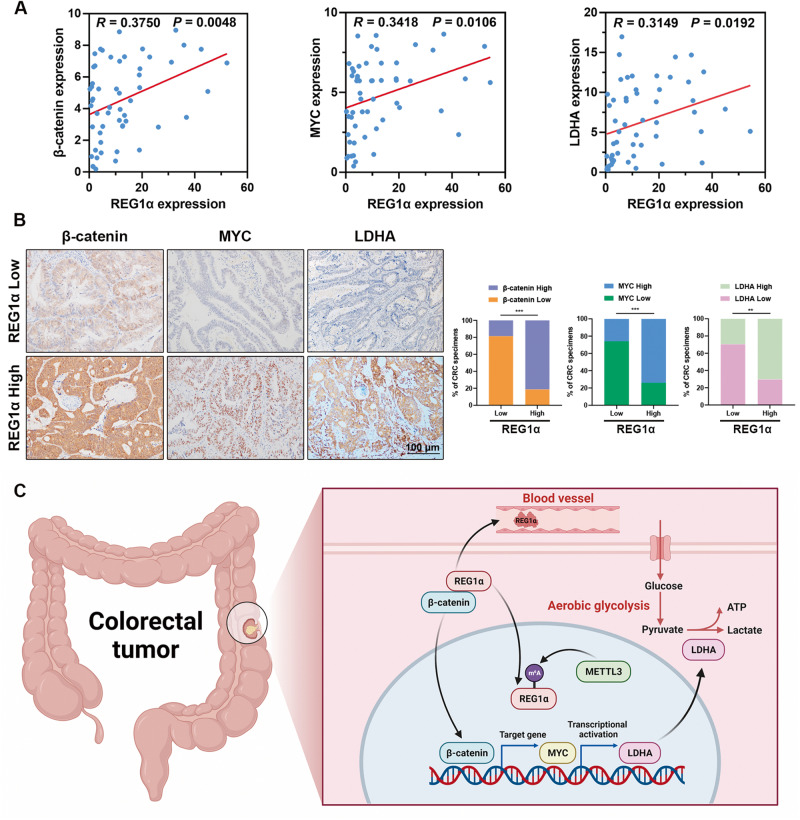
REG1α positively correlates with β-catenin, MYC and LDHA expression in CRC samples. **A** The mRNA correlation between REG1α and β-catenin, MYC and LDHA expression in a cohort of 55 CRC patients were analyzed by qRT-PCR. **B** The representative images of immunohistochemical staining of β-catenin, MYC and LDHA expression from CRC samples with different levels of REG1α. **C** Schematic diagram showing the proposed molecular mechanisms of m^6^A/REG1α/β-catenin/MYC signaling in CRC. Scale bar = 100 μm. Significant differences were shown by **P < 0.01 and ***P < 0.001.

To illustrate the clinical correlation between REG1α and β-catenin, MYC and LDHA in CRC sample, we analyzed their expression levels in our cohort of 55 CRC patients by RT-PCR. As shown in Fig. 8A, the elevated REG1α in CRC tissues was positively related to the increased β-catenin (Spearman’s rank correlation coefficient, *R* = 0.3750, *P* = 0.0048), MYC (Spearman’s rank correlation coefficient, *R* = 0.3418, *P* = 0.0106) and LDHA (Spearman’s rank correlation coefficient, *R* = 0.3149, *P* = 0.0192). We next assessed the relationships between REG1α and β-catenin, MYC, LDHA expression by immunohistochemistry in 54 CRC samples. Representative images of the intense staining of REG1α and β-catenin, MYC, LDHA from the same patients were shown in Fig. 8B, indicating the tight correlations between REG1α and its downstream targets. Collectively, our findings indicated that REG1α, upregulated by METTL3 via m^6^A modification, regulated CRC progression via MYC/LDHA mediated glycolysis (Fig. 8C).

## Discussion

The human REG family proteins are known to be involved in cell proliferation of gastrointestinal cells, cardiovascular cells, hepatic cells, and neuronal cells [22–24]. Recently, intensive research unveiled that REG1α is remarkably highly expressed in various malignant tumors, including CRC. Geng et al. reported that REG1α could be used as a marker of prognosis and recurrence in bladder cancer, and downregulation of REG1α reduced tumor growth, migration, invasion and angiogenesis [25]. Nasr et al. found that REG1α was highly expressed in both tubular casts and tumor cells in a patient with pancreatic acinar-neuroendocrine carcinoma [26]. Besides, in primary gastric epithelial cells, helicobacter and gastrin stimulated the expression of REG1α and accelerated the progression of gastric cancer [27]. In human pancreatic cancer cells, core 3 synthase significantly suppressed tumor growth and metastasis through downregulating the expression of several genes including REG1α [28]. However, almost all previous studies only examined the expression of REG1α in cancer and its correlation with clinical and pathological features. Few studies explored the biological role of REG1α in CRC development at the molecular level. In this study, we firstly uncovered that REG1α played a pivotal role in regulating CRC cell proliferation, migration both in vitro and in vivo, and the upregulated REG1α in CRC patients was significantly correlated with lymph node metastasis, advanced tumor stage and worse prognosis. Intriguingly, serum REG1α achieved AUC values of 0.7806, indicating its promising potential as a non-invasive biomarker in distinguishing CRC patients and healthy controls.

To date, the reprogramming of energy metabolism has been listed as a prominent hallmark of cancer [29]. Cancer cells conducted high levels of glycolysis even in the condition of ample oxygen, which was essential for cell survival and metastasis [30]. Thus, it has been suggested that targeting glucose metabolism may provide a selective mechanism which specifically impedes the proliferation and invasion of cancer cells [31]. Mounting evidence confirmed that several potential candidates were overexpressed in certain cancers and contributed to glycolysis, including FUBP1, HIF-1α, and CD47 [32]. However, studies concerning the identification of potential candidates regulating glycolysis in CRC cells are still lacking. Herein, our study found that forced expression of REG1α significantly coordinates the Warburg effect, as manifested by the increased glycolysis uptake, lactate production, ATP and ECAR level of CRC cells. Inhibition of REG1α markedly decreased the level of glycolysis under basal conditions, and the glycolytic capacity. To the best of our knowledge, this is the first study reported that REG1α is a novel glycolytic regulator, which exerted its tumor-promoting functions through evoking aerobic glycolysis of cancer cells. Elucidating the energy metabolic-related functions of REG1α will provide a better understanding of the modulatory mechanism concerning CRC cell metabolism. Besides, recent researches also highlighted the essential role of gut microbiota in CRC tumorigenesis. Some bacteria, such as *Escherichia coli*, *Bacteroides fragilis,* and *Peptostreptococcus anaerobius* could facilitate CRC progression by directing DNA damage, activating Th17 cells or inducing cholesterol synthesis [33]. However, the crosstalk between REG1α and gut microbiome remain to be investigated in further research.

MYC is a notorious marker in the development of multiple cancers. Previous studies have demonstrated that MYC has participated in many biological processes, such as cell proliferation, cell cycle, energy metabolism, and apoptosis [34]. In human prostate cancer, MYC drove glucose metabolism via the suppression of TXNIP (a potent negative regulator of glucose uptake), which was predominantly dependent on the glutaminase-MondoA axis [35]. Recent evidence strongly suggested that the oncogenic effector MYC was highly expressed in many human malignancies. Meanwhile, MYC-dependent metabolic reprogramming characterized by heightened glycolysis, enhanced fatty acid, and nucleotide synthesis was critical for tumorigenesis [36]. Tang et al. have identified GLCC1, a lncRNA that was remarkably overexpressed under glucose starvation in CRC cells and supported cell proliferation and survival by stabilizing MYC from ubiquitination and enhancing glycolysis [37]. In addition, overexpression of lncRNA MEG3 significantly induced MYC degradation by ubiquitination and inhibited MYC-related target genes in the glycolysis pathway, thus effectively suppressing aerobic glycolysis in CRC cells [38]. We here showed that the prominently upregulated MYC was under the control of REG1α in CRC cells. Interestingly, by using MYC and LDHA inhibitors, we found that both MYC and LDHA were indispensable for REG1α-induced increasement in glycolysis. Furthermore, ChIP assay revealed that MYC activated LDHA transcription by directly binding to its promoter regions, and this phenomenon was exacerbated under REG1α overexpression in CRC cells.

As a remarkable dysregulated oncogene, MYC transcription could be activated by the Wnt/β-catenin signaling pathway. It was reported that in human breast cancer, Wnt/β-catenin mediated the increment of cell aerobic glycolysis [39]. In addition, the activation of Wnt/β-catenin signaling triggered its downstream target PDK1, thereby promoting glycolysis process and angiogenesis [40]. To determine the precise mechanisms underpinning the REG1α-mediated MYC upregulation, CRC cells were subjected to various pathway inhibitors and we noticed that only XAV-939 (a specific inhibitor of Wnt/β-catenin signaling) resulted in the decrease of MYC expression. Besides, our GSEA results revealed that the Wnt signaling pathway was prominently positively correlated with REG1α in CRC patients, suggesting that REG1α may facilitate MYC expression via boosting Wnt/β-catenin signaling in CRC. In the current research, we found that administration of XAV-939 obviously attenuated the translocation of β-catenin from the cytoplasm to nucleus and a series of malignant properties, including glycolysis, cell proliferation, migration and invasion mediated by exogenous REG1α. Thus, our data revealed that REG1α facilitated MYC-mediated glycolysis of CRC via promoting the activation of Wnt/β-catenin signaling and broadened the knowledge regarding the regulatory mechanism of MYC in CRC.

N^6^-methyladenosine (m^6^A), as one of the most prevalent epigenetic modifications on RNA, is closely associated with human carcinogenesis [41, 42]. Accumulating evidence has underscored the m^6^A writer METTL3, exhibited an oncogenic potentiality in CRC tumorigenesis recently, wherein it accelerated tumor growth and metastasis through suppressing the expression of YPEL5 in an m^6^A-YTHDF2-dependent manner [43]. Besides, Li et al. also revealed that METTL3 maintained SOX2 expression in CRC through an m^6^A-IGF2BP2-dependent manner. Knocking down METTL3 drastically restrained CRC cell self-renewal, stem cell frequency in vitro and inhibited tumorigenesis and metastasis in both patient-derived xenograft (PDX) and cell-based models [44]. Previous studies have reported that METTL3, as the top essential m^6^A regulatory enzyme in CRC development, could induce GLUT1 translation and promote glucose metabolism in an m^6^A-dependent manner [45]. As REG1α strikingly promotes glycolysis in CRC cells, we wonder whether there exists an association between REG1α and METTL3. Intriguingly, we found that S-adenosylmethionine synthesis inhibitor DAA treatment stimulated the mRNA levels of REG1α, and forced expression of METTL3 markedly upregulated expression of REG1α at both the mRNA and protein level and prolonged the lifetime of the REG1α mRNAs. Besides, deletion of REG1α in METTL3-overexpressed cells effectively reversed METTL3-drives glycolytic metabolism and malignant phenotypes. Our study provides the first evidence that REG1α is controlled by METTL3-mediated m^6^A modification, which partially account for the elevated expression of REG1α in CRC.

In summary, our results delineated that upregulated REG1α predicted lymph node metastasis, advanced TNM stage, and poor prognosis of CRC patients. A series of functional experiments suggested that REG1α serves as an oncogene in CRC by accelerating cell growth, metastasis, and glucose metabolism in an MYC-dependent manner. Mechanistically, MYC was triggered by the Wnt/β-catenin pathway and directly bound to LDHA to enhance aerobic glycolysis. Moreover, m^6^A epigenetic modification mediated by METTL3 increased and stabilized REG1α expression. Our research provided a scientific basis for targeting the REG1α/β-catenin/MYC/LDHA axis as a feasible therapeutic approach for CRC patients.

## Material and methods

### Patients and specimens

Three cohorts containing 152 fresh CRC tissues and their paired non-cancerous tissues, 62 CRC and 58 healthy donor’s serum samples and 68 paraffin-embedded CRC tissue were collected from The First Affiliated Hospital of Zhengzhou University. All patients involved in this study received no treatment before surgery and were given written informed consent. The experimental protocol was approved by the ethics committee of the First Affiliated Hospital of Zhengzhou University. CRC cases were classified based on the eighth edition of the AJCC TNM staging system.

### Enzyme-linked immunosorbent assay (ELISA)

Whole blood samples were collected from CRC patients prior to surgery and centrifugated at 1000 × *g* for 15 min to extract serum. Serum REG1α levels in CRC patients and healthy controls were analyzed using Human REG1α ELISA Kit (NBP2-82145, Novus Biologicals). Each sample was tested in quadruplicate and incubated with Biotinylated Detection Ab for one hour at 37 °C. REG1α concentration was read at optical density (OD value) 450 nm and calculated based on standard curves.

### Cell culture

Normal human colonic epithelial cell line NCM460 and FHC, and a panel of human CRC cell lines, including DLD1, SW480, SW620, and HCT116 cells were purchased from the American Type Culture Collection (ATCC) and preserved in our laboratory. NCM460 and FHC cells were grown in RPMI1640 medium and all CRC cells were cultured in high glucose DMEM medium supplemented with 10% Fetal Bovine Serum (FBS, VivaCell, Shanghai, China) and 1% penicillin/streptomycin (Haixing Biosciences, China) in a humidified atmosphere at 37 °C with 5% CO_2_. Cell culture dishes and plates were purchased from NEST Biotechnology (Wuxi, China). All cell lines have been authenticated by STR and confirmed without mycoplasma contamination. Signaling pathway inhibitors PD98059 and SB431542 were purchased from Selleckchem (Shanghai, China). XAV-939 was purchased from TargetMol (USA). Stattic was purchased from Med Chem Express (MCE, China).

### RNA extraction and qRT-PCR

Total RNA from CRC tissues and different cell lines was extracted using RNAiso Plus Reagent (Takara Biotechnology, China). The first-strand cDNA was synthesized by using PrimeScript RT Master Mix (Takara Biotechnology, China). Then real-time PCR was conducted by using qPCR SYBR Green Master Mix (Cat11202, Yeasen, Shanghai, China) on Applied Biosystems 7500 instrument. The relative expression was normalized to β-actin or GAPDH (Sangon Biotech, Shanghai, China) by the 2^−ΔΔCt^ method. Detailed primer sequences of this study were listed in Supplementary Table S2.

### Immunohistochemistry staining

Slides were heated in a solution of pH 6.0 sodium citrate for 20 min to undergo microwave antigen retrieval after deparaffinization and rehydration. Then the sections were kept in 0.6% H_2_O_2_ to quench endogenous peroxidase. Afterward, the sections were incubated with the corresponding primary antibody at 4 °C overnight. After washing, the slides were incubated with horseradish peroxidase (HRP)-conjugated secondary antibody. Finally, the slides were stained with DAB (LABLEAD Inc.) and visualized by microscopy.

### Western blot

Cell lysates and human CRC tissues were lysed by ice-cold RIPA buffer (Beyotime Biotechnology, China) containing protease and phosphatase inhibitors (New Cell & Molecular Biotech, China). BCA protein assay kit (Novoprotein, Shanghai, China) was used to determine the concentration of total proteins. An equal amount of 20–50 micrograms of whole cell lysates or total tissue lysates were separated on 8–12% SDS-PAGE gel, followed by transfer on to polyvinylidene fluoride (PVDF) membrane (Millipore, Germany). Then the membranes were blocked in 5% BSA for 1 h and then incubated with the corresponding primary antibodies at 4 °C overnight. All of the antibodies used in this study were shown in Supplementary Table S3.

### Vectors construction

The cDNA encoding human REG1α was amplified by PCR and cloned into the lentiviral expressing vector pCDH-CMV-MCS-EF1α-puro as described previously, and the empty plasmid was utilized for the control group [46]. For stable REG1α knockdown cells, short-hairpin RNA was annealed and inserted into the pLKO.1-neo vector, while a scramble shRNA was used as the negative control. All constructed plasmids were verified by DNA sequencing (GENEWIZ, Suzhou, China) and transfected into 293T cells along with psPAX2 and pMD2.G using Lipofectamine 3000 (Invitrogen, USA). Virus supernatants were collected at 48 h post-transfection and infected with CRC cells combined with polybrene. Puromycin or neomycin were added to CRC cells for consecutive seven days to acquire stable REG1α overexpression or knockdown cells.

### Cell proliferation and colony-formation assay

CRC cell viability was determined by using Cell Counting Kit-8 (CCK-8, Dojindo, Japan) reagent. One thousand cells per well with a complete culture medium were seeded into a 96-well plate with five repeats. After incubation for 24 h, 10 μL CCK-8 reagents were added to cells and incubated for another 2 h in the dark at 37 °C. A multifunctional microplate reader was used to detect the absorbance at 450 nm for five consecutive days. Cell proliferation was also determined by the EdU incorporation assay. Ten thousand stable knockdown or overexpressing REG1α were stained with 50 μM EdU medium at 37 °C for 2 h, then fixed by 4% paraformaldehyde and incubated with Apollo567 staining buffer and finally observed under the fluorescence microscope. For the colony-formation assay, ~500 cells per well were seeded into the 35 mm dishes and cultured with a complete medium for ten days. The culture medium was changed at 3 day intervals. Finally, the cells were fixed with 4% paraformaldehyde and stained with 0.1% crystal violet.

### Cell apoptosis and cycle analysis by flow cytometry

Annexin V-FITC apoptosis detection kit (APExBIO, Houston, USA) was used to detect cell apoptosis. After starving for 48 h, samples were washed and resuspended at a density of 1 × 10^6^ cells/mL in the staining buffer, followed by the analysis with a FACSCanto flow cytometer (BD Bioscience). For cell cycle analysis, cells were fixed with 70% ethanol at 4 °C overnight, then washed and resuspended in 500 μL PI/RNase buffer (BD Bioscience), incubated at room temperature for 15 min, and analyzed by the flow cytometer.

### Cell migration and invasion assay

Chamber inserts were scrawled with Matrigel and dried at 37 °C for 6 h. For cell migration and invasion assays, experimental procedures were performed as previously described [47]. 1 × 10^5^ CRC cells with the serum-free medium were added into the upper chamber, and complete medium supplemented with 20% FBS was added into the bottom chamber as chemoattractant. Following incubation at 37 °C for 24 h, the migrated or invaded tumor cells were fixed with 4% paraformaldehyde, stained with 0.1% crystal violet at room temperature, and dried in the air. Cell migration ability was assessed at least three independent visual fields were photographed each time.

### Glycolysis stress test

The extracellular acidification rate (ECAR) and oxygen consumption rate (OCR) were determined by a Seahorse XF96 Analyzer Glycolysis according to the manufacturer’s instruction (Seahorse Bioscience). CRC cells were seeded in a XF96-well plate with the density of 10^4^ per well with REG1α overexpression or knockdown. As glycolysis occurs, the XF Analyzer can directly detect the acidification from the culture medium around CRC cells and reported as the ECAR. Following this, a saturating concentration of glucose, oligomycin and 2-deoxyglucose (2-DG) were added to the cells at the indicated time points. In the meanwhile, the ECARs were detected. For the assessment of OCR, oligomycin, Trifluoromethoxy carbonylcyanide phenylhydrazone (FCCP) and Rotenone were sequentially added to the cells.

### Measurement of glucose, lactate, and ATP

The glucose consumption assay was performed using a commercial glucose assay kit (Sigma-Aldrich, USA), and the glucose consumption levels were calculated by the difference value of measured glucose concentration in the medium from the beginning. Lactate production was quantitated by using a colorimetric lactate assay kit (BioVision, USA) according to the manufacturer’s instructions. SW620 and HCT116 cells with REG1α overexpression or knockdown were cultured in the lactate assay buffer comprising lactate probes and enzymes. Finally, the optical density was measured at 570 nm. Adenosine triphosphate (ATP) content was evaluated using an ATP Content assay kit (Beijing Solarbio Science & Technology Co., Ltd., China) following the manufacturer’s instructions.

### Co-immunoprecipitation (Co-IP) assay

CRC cells were cleaved on ice with lysis buffer for IP (Beyotime, China) containing protease inhibitor. Then the protein concentrations were detected by a BCA Protein Assay Kit and 50 μg protein was extracted from each sample. REG1α or β-catenin antibody for co-IP was added to the sample and the cell lysate/antibody mixture was gently rotated at 4 °C on a rotator overnight. The next day, a total of 20 μL Protein A/G magnetic beads (Thermo Scientific, USA). were added to each sample and incubated at 4 °C for 2 h with rotation. The beads were washed three times using 800 μL IP lysis buffer and after centrifugation, the supernatant was discarded and 20 μL 2 × loading buffer was added to the beads. Finally, the lysed immune complexes were fractionated by SDS/PAGE and analyzed by western blot.

### Chromatin immune-precipitation (ChIP) assay

A commercial ChIP kit (Millipore, USA) was used for ChIP assay. Briefly, CRC cells with indicated treatment were fixed with 1% formaldehyde at indoor temperature for 10 min. After the immobilization and crosslinking of DNA and protein, the samples were broken by ultrasonic processor into 200–500 bps. Next, negative control rabbit anti-IgG or anti-MYC antibody was added to the supernatant, respectively. Agarose/Sepharose was used to precipitate the endogenous DNA protein complex. Then the samples were washed, de-crosslinked, and the DNA fragment was recovered by phenol/chloroform extraction and purification. The purified DNA was quantitatively pulled down using qRT-PCR.

### Methylated RNA immunoprecipitation (MeRIP)

The indicated CRC cells were seeded in 10 cm dishes and dissociated in Polysome lysis buffer with protease inhibitor cocktails and RNase inhibitors. Cellular lysates were immunoprecipitated to anti-m^6^A or IgG antibody, which was linked to Pierce protein A/G magnetic beads (Thermo Scientific, USA), and the mixture was rotated at 4 °C overnight. The samples were washed six times using lysis buffer and digested at 55 °C with proteinase K, modification of m^6^A towards REG1α genes was determined by RT-PCR analysis.

### Luciferase reporter assay

TOP/FOP FLASH reporter system was employed to measure the activity of Wnt/β-catenin pathway upon inhibition or overexpression of REG1α. Briefly, CRC cells were seeded in 24-well plates, and after culturing for 24 h, then cells were co-transfected with TOPflash/FOPflash reporter plasmids and Renilla luciferase plasmids using Lipofectamine 3000 (Invitrogen, USA). Cells were lysed after 24 h of transfection and the relative luciferase activity was assessed by Luciferase Assay System (Promega, USA) and calculated based on the Renilla luciferase activity. To determine the effect of OE-REG1α or shMYC on LDHA promoter activity, genomic human LDHA promoter with or without predicted mutation binding sites was cloned into the pGL3 vector and co-transfected with Renilla luciferase reporter plasmid into indicated CRC cells. At 24 h after transfection, the luciferase activity in different groups were measured by a Dual-Luciferase Reporter System (Promega, USA). Fragments of the REG1α 3’UTR with wild-type m^6^A motifs or mutant m^6^A motifs were inserted into the pmirGlo dual-luciferase expression vector (Promega, USA), and the subsequent procedures were manipulated as above.

### Animal studies

All animal experiments were performed under the approval of the Institutional Animal Care and Use Committee of Zhengzhou University. Six-week-old female Balb/c nude mice (Shanghai SLAC Laboratory Animal, China) were fed with water or diet with free access. The mice were randomly divided into four groups and were subcutaneously injected with equal amounts of OE-REG1α and shREG1α-1 cells and their corresponding control cells. Tumor growth was monitored every five days, tumor volume was measured with a caliper and recorded. After observing for 35 days, the mice were euthanized to isolate the tumors. For in vivo lung metastases model, a total of 2 × 10^6^ CRC cells with REG1α-overexpression or REG1α-knockdown were injected into nude mice via lateral tail vein. Six weeks after injection, the mice were sacrificed and the lung tissues were collected. The nodules were counted and images were taken. The lung metastases were assessed using hematoxylin and eosin (H&E) staining. For the intrasplenic inoculation model, the mice were first inhalation anesthetized with 2.5% isoflurane. After confirming the success of anesthesia, sterile animal surgical instruments were used to make a small incision in the left abdomen of mice to expose the spleen. Then 2 × 10^6^ tumor cells were slowly injected into the spleen using a 1 mL syringe. Once the intrasplenic injection had been completed, the spleen was carefully returned to the abdomen of the mouse and the muscle and skin were sewed. Then the mice were kept under observation until complete recovery. On day 49, the mice were sacrificed, the liver were removed and fixed in 10% formaldehyde for the following H&E staining to evaluate the numbers of metastatic nodules on the liver.

### Statistical analysis

Experimental data were presented as mean ± standard deviation (SD) and analyzed by GraphPad Prism 9 software. Differences between different groups were analyzed by using paired or unpaired Student’s *t*-test. Overall survival curves were plotted using the Kaplan-Meier method. Data used for statistical analysis were from at least three representative independent experiments. *P < 0.05, **P < 0.01, and ***P < 0.001 were considered to denote statistical significance.

## Supplementary information

Reproducibility checklist

Supplemental information

Full and uncropped western blots

## Data Availability

The data used to support the findings of this study are available from the corresponding author upon request.
