# Supplementary material for: N6-methyladenosine modification of REG1α facilitates colorectal cancer progression via β-catenin/MYC/LDHA axis mediated glycolytic reprogramming
Source: Cell Death Dis. 2023 Aug 25;14(8):557. doi: 10.1038/s41419-023-06067-6 (PMC10457312; doi:10.1038/s41419-023-06067-6)
Supplement: Supplementary file 2 — Supplemental information [file 41419_2023_6067_MOESM2_ESM.pdf]

Supplementary Figures

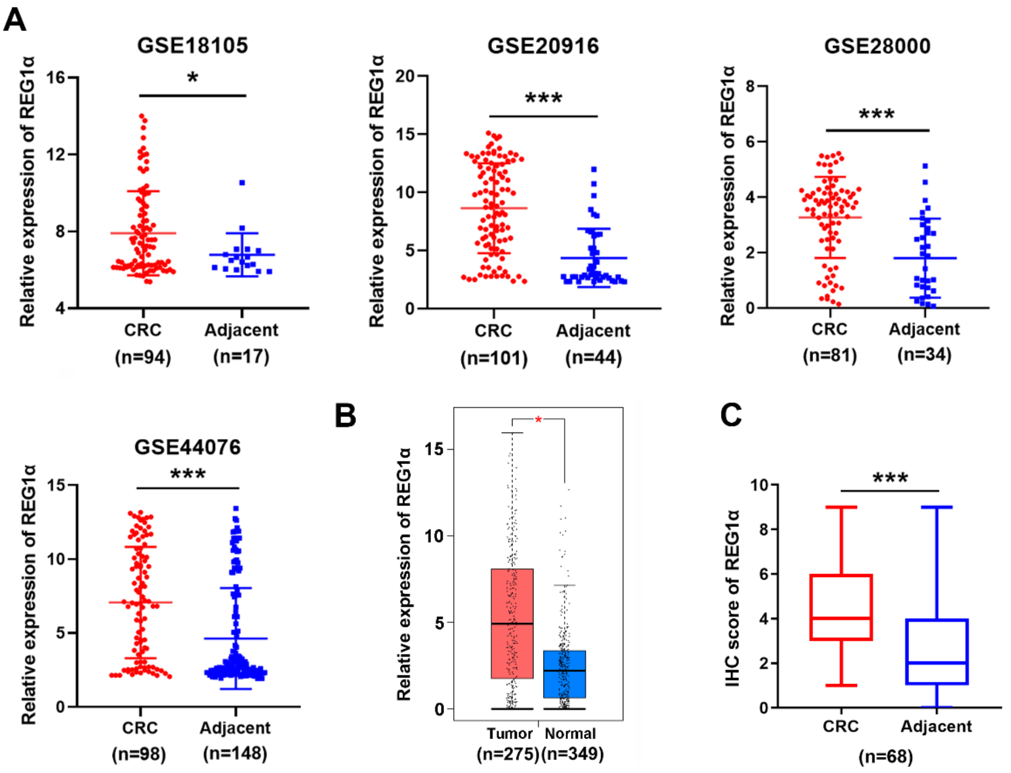

Fig. S1 REG1α is significantly elevated in colorectal cancer.

A The expression pattern of REG1α mRNA level in CRC patients based on the different GEO datasets. **B** Expression profiles of the mRNA level of REG1α in colon adenocarcinoma tissues and normal colorectal tissues according to the TCGA data based on GEPIA website. **C** Statistical analysis of immunohistochemical staining score of REG1α in CRC samples and paired adjacent tissues. Significant differences were shown by *$P < 0.05$ and ***$P < 0.001$.

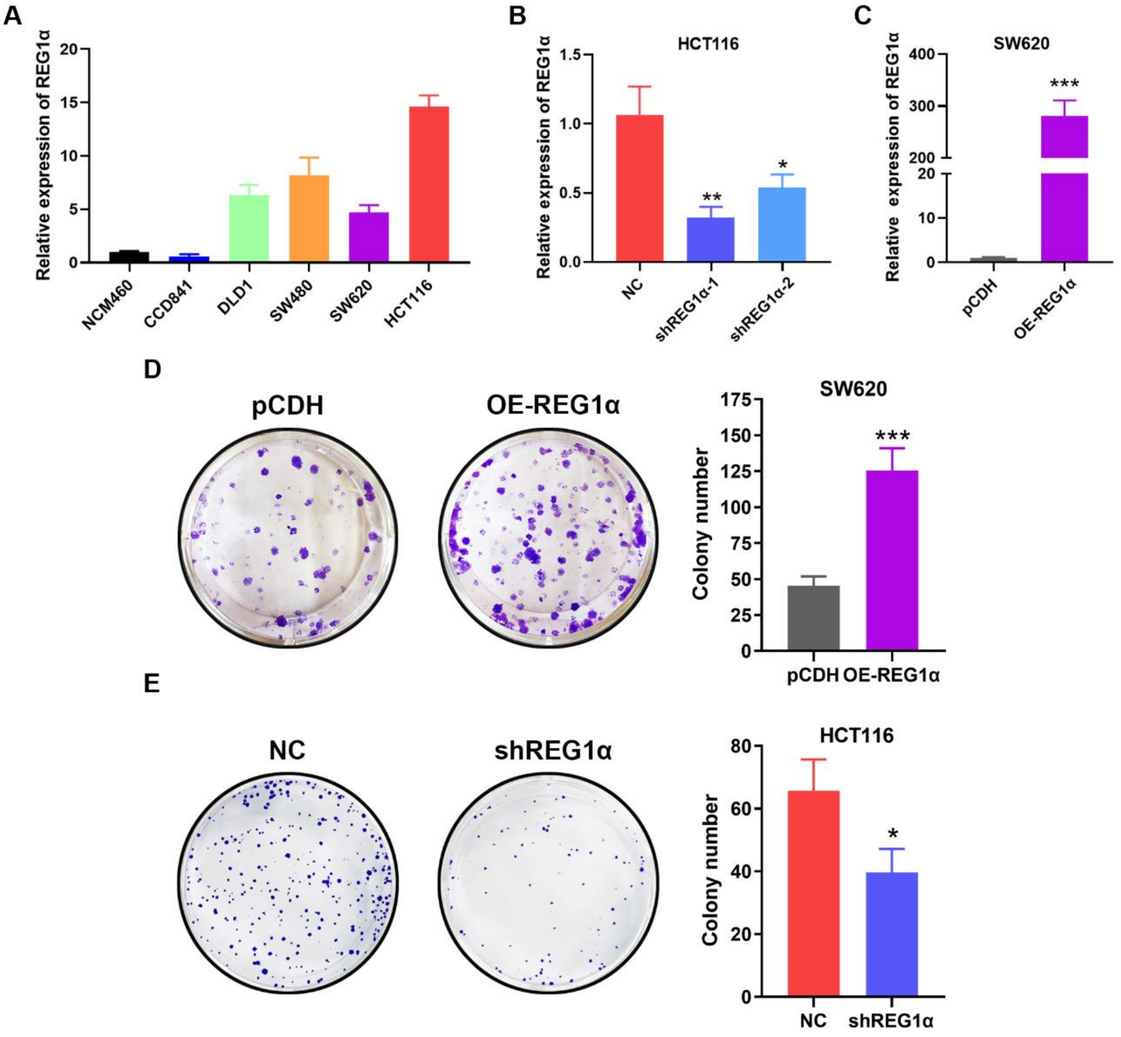

Fig. S2 Generation of stable REG1α-knockdown and overexpression plasmids in CRC cells.

A Expression of REG1α in human colon epithelial cells and colorectal cancer cell lines was detected by qRT-PCR. **B-C** qRT-PCR showing the knockdown and overexpression efficiencies of REG1α in HCT116 and SW620 cells. **D-E** Effects of ectopic expression or knockdown of REG1α on the abilities of colony-forming in CRC cells. Significant differences were shown by *$P < 0.05$, **$P < 0.01$, and ***$P < 0.001$.

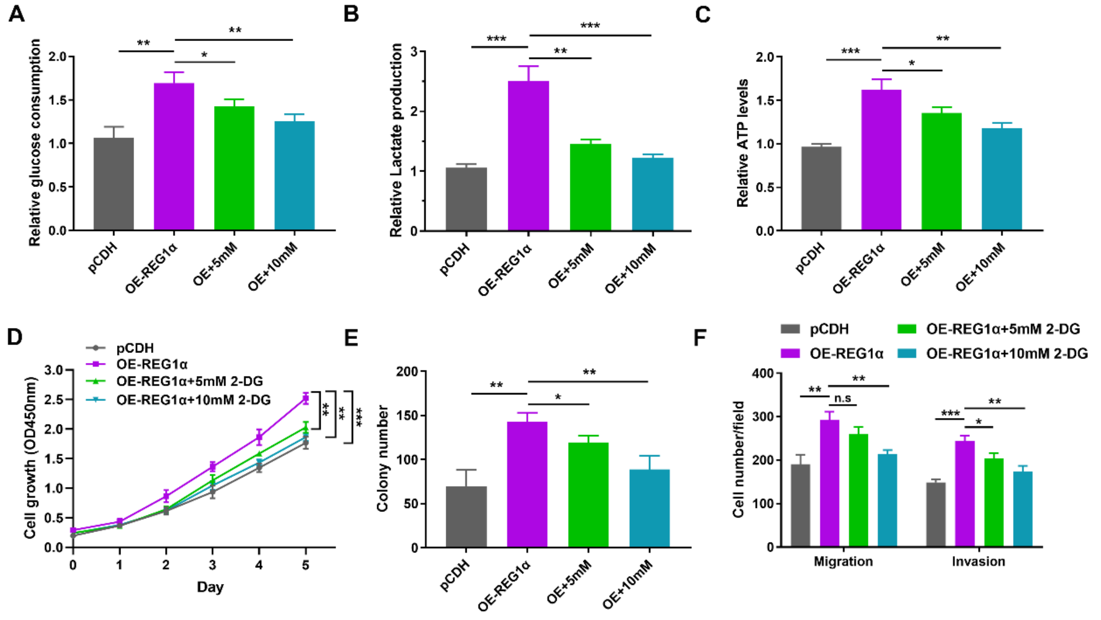

Fig. S3 Inhibition of glycolysis with 2-DG reverses the tumor-promoting effects of REG1α in CRC.

A-C Relative glucose consumption **(A)**, lactate concentration **(B)**, ATP production **(C)** were measured in control group and REG1α-overexpression cells with different concentrations of 2-DG. **D-E** CCK-8 **(D)** and colony-formation **(E)** assays were used to detect the role of 2-DG in CRC cell viability. **F** Effects of 2-DG on the migration and invasion of SW620 cells with REG1α overexpression. Significant differences were shown by $*P < 0.05$, $**P < 0.01$, and $***P < 0.001$.

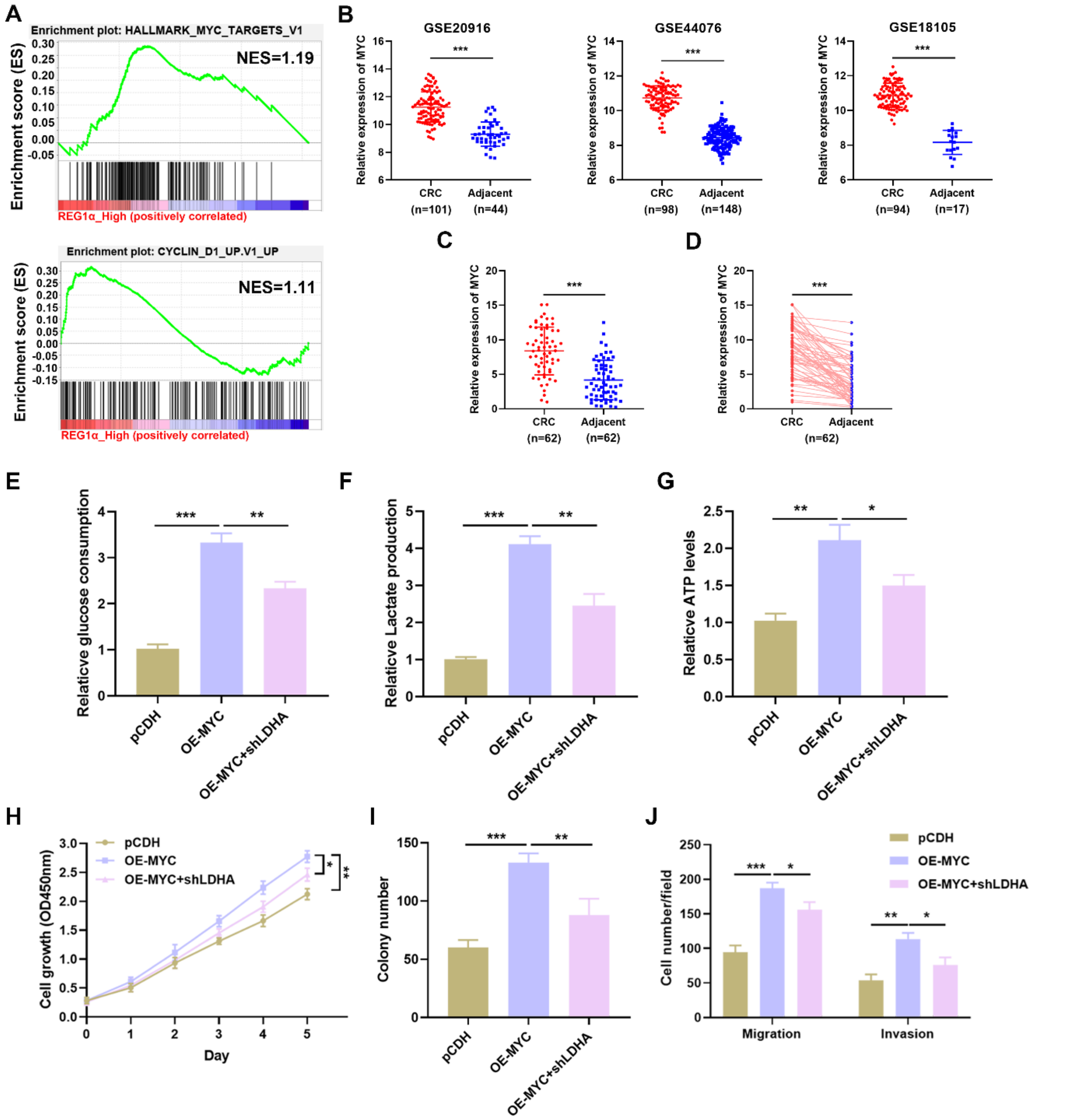

Fig. S4 LDHA is responsible for MYC-induced glycolysis of CRC cells.

A GSEA analysis in CRC patients showed that the upregulated MYC and CCND1 were strongly associated with high REG1α expression. NES, normalized enrichment score. **B** The colorectal tissues and CRC gene expression of MYC were analyzed in different GEO datasets. **C-D** qRT-PCR (n=62) analysis of MYC level in CRC tissues and paired normal colorectal tissues. MYC was expressed relative to the normal controls and normalized to β-actin. **E-G** The glycolysis-related phenotypes in CRC cells with MYC overexpression, MYC

overexpression plus shLDHA or control cells were determined. **H-J** Specific shRNA targeting LDHA attenuated tumor proliferation **(H)**, colony-formation **(I)** and mobility **(J)** induced by MYC overexpression in CRC cells. Significant differences were shown by *$P < 0.05$, **$P < 0.01$, and ***$P < 0.001$.

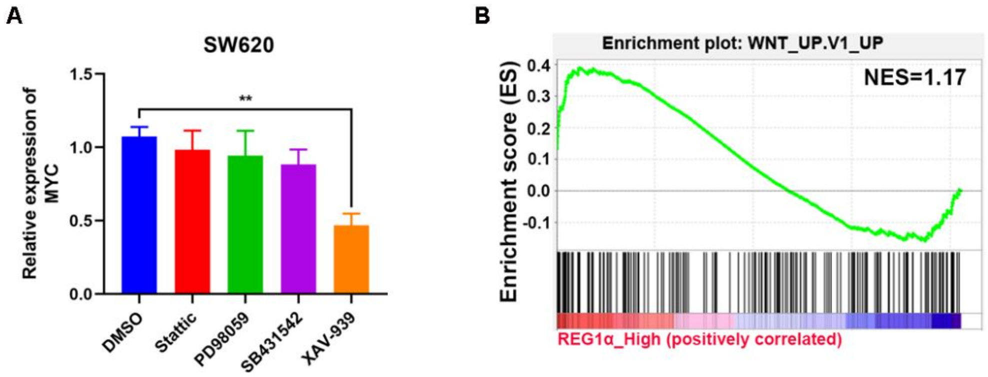

Fig. S5 Wnt/β-catenin signaling pathway is required for REG1α-induced MYC upregulation.

A qRT-PCR analysis of MYC mRNA level in SW620 cells with REG1α overexpression after treatment with different pathway inhibitors for 48 hours. **B** GSEA analysis indicated that a significant change of Wnt/β-catenin signaling was correlated with REG1α expression. NES, normalized enrichment score. Significant differences were shown by $^{**}P < 0.01$.

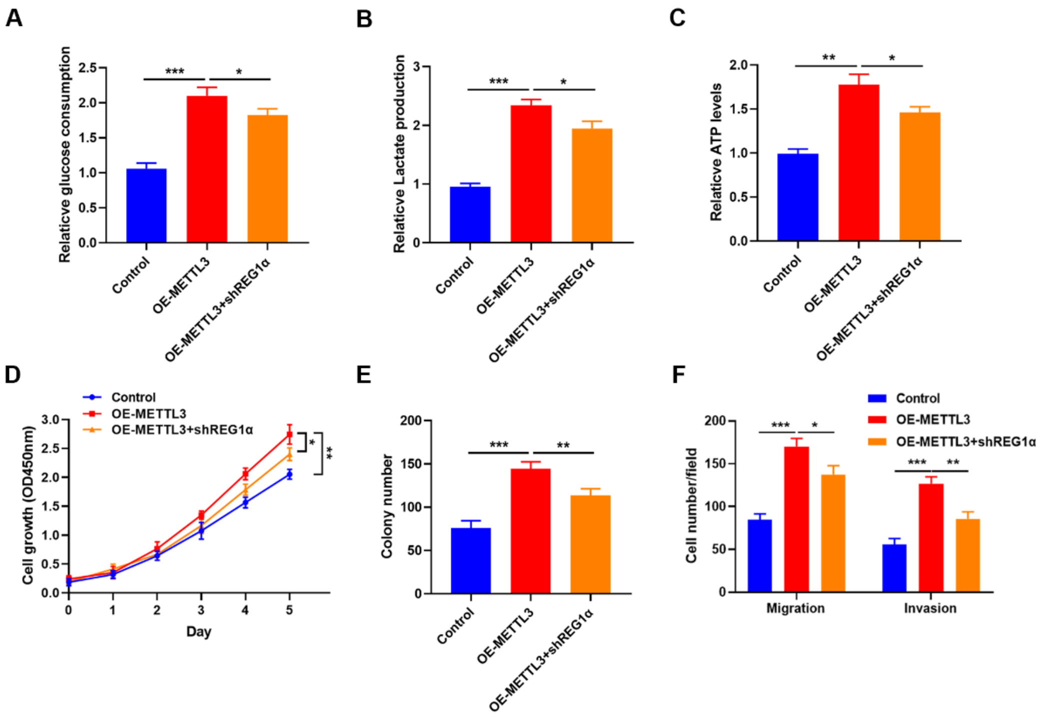

Fig. S6 Inhibition of REG1α suppressed METTL3-mediated malignant behaviors.

A-C Statistical analysis of glucose consumption **(A)**, lactate concentration **(B)** and ATP production **(C)** of METTL3-overexpressing HCT116 cells transfected with shREG1α or their corresponding controls. **D-E** REG1α influences cell proliferation **(D)** and colony-forming abilities **(E)** in METTL3-overexpressing HCT116 cells. **F** The effects of REG1α or METTL3 on the migration and invasion abilities of HCT116 cells were detected by Transwell assays. Data from as least three independent experiments and presented as the mean ± S.D.. Significant differences were shown by *$P < 0.05$, **$P < 0.01$, and ***$P < 0.001$.

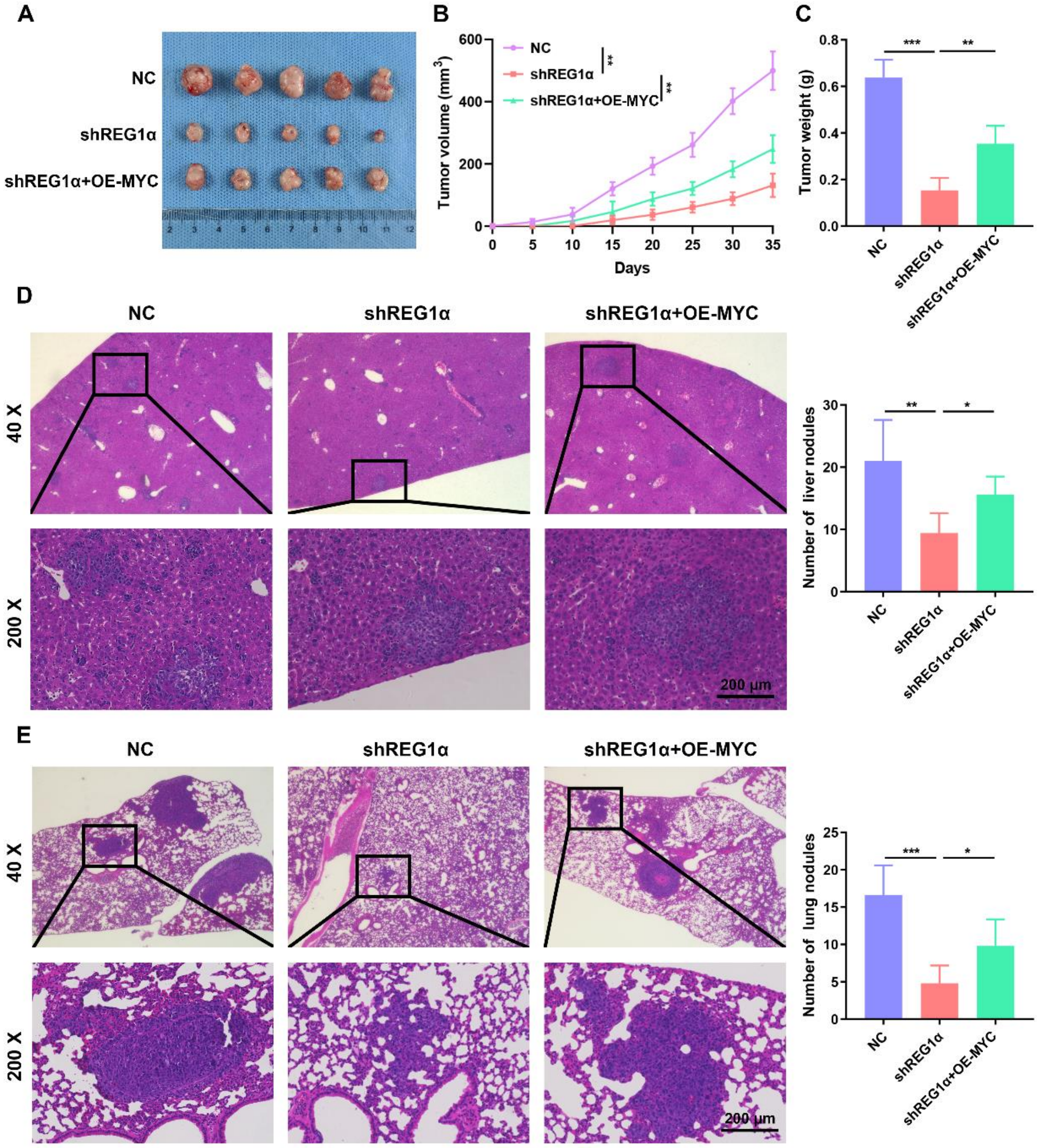

Fig. S7 REG1α knockdown retards tumor growth and inhibits tumor metastasis of CRC cells in vivo.

A Representative photos of tumors isolated from subcutaneous xenografts injected with negative control (NC), shREG1α, and shREG1α with OE-MYC cells. **B-C** Subcutaneous xenograft tumor growth **(B)** and weights **(C)** in nude mice (5 per group) were measured and compared in HCT116 cells (NC vs. shREG1α vs. shREG1α plus OE-MYC). **D** Representative H&E staining images of metastatic liver nodules in nude mice injected with negative control

and REG1α silenced HCT116 cells with or without MYC overexpression. **E** The effects of REG1α knockdown on CRC cell metastasis were also determined by experimental lung metastasis model. Representative H&E staining of pulmonary metastatic nodules were shown. Scale bar = 200 μm. Significant differences were shown by $*P < 0.05$, $**P < 0.01$, and $***P < 0.001$.

Table S1. Correlation between mRNA expression of REG1α and clinical characteristics in 152 CRC patients.

Variables	No. of patients	REG1α expression		*P*-value
		High group (n=76)	Low group (n=76)	
Gender				
Male	80	39	41	0.745
Female	72	37	35	
Age				
<65	86	44	42	0.743
≥65	66	32	34	
Tumor grade				
G1+G2	102	46	56	0.084
G3+G4	50	30	20	
Tumor invasion				
T1+T2	74	42	32	0.105
T3+T4	78	34	44	
Size (cm)				
<5	96	51	45	0.313
≥5	56	25	31	
Lymph node status				

N0	68	25	43	0.003**
N1+N2	84	51	33	
TNM stage				
I+II	66	24	42	0.003**
III+IV	86	52	34	

TNM: tumor-node-metastasis, ***P* < 0.01.

Table S2. The primers used in this study for RT-PCR analysis.

Genes	Forward primer (5'-3')	Reverse primer (5'-3')
REG1α	ACCAGCTCATACTTCATGCTG	CCAGGTCTCACGGTCTTCAT
MYC	GGCTCCTGGCAAAAGGTCA	CTGCGTAGTTGTGCTGATGT
ALDOB	CCACCGTAACAGCTCTCCAC	CACTCATGCCACCAGACAAAA
GLUT1	TCTGGCATCAACGCTGTCTTC	CGATACCGGAGCCAATGGT
GLUT2	GCTGCTCAACTAATCACCATGC	TGGTCCCAATTTTGAAAACCCC
HK2	TTGACCAGGAGATTGACATGGG	CAACCGCATCAGGACCTCA
PDK1	GGATTGCCCATATCACGTCTTT	TCCCGTAACCCTCTAGGGAATA
LDHA	ATGGCAACTCTAAAGGATCAGC	CCAACCCCAACAACTGTAATCT
PKM2	ATAACGCCTACATGGAAAAGTGT	TAAGCCCATCATCCACGTAGA
ENO2	AGGTGCAGAGGTCTACCATAC	AGCTCCAAGGCTTCACTGTTC
G6PDH	GCAGAGCACAAGGATCAGTTC	GGCAGCTACTGTTGATGTTGC
β-actin	GCACAGAGCCTCGCCTT	CCTTGCACATGCCGGAG
MMP7	GAGTGAGCTACAGTGGGAACA	CTATGACGCGGGAGTTTAACAT
MMP9	TGTACCGCTATGGTTACACTCG	GGCAGGGACAGTTGCTTCT
CCND1	CAATGACCCCGCACGATTTC	CATGGAGGGCGGATTGGAA
METTL3	AAGCTGCACTTCAGACGAAT	GGAATCACCTCCGACACTC

Table S3. The antibodies used in this study.

Antibodies	Catalog#	Source
anti-REG1α	15850-1-AP	Proteintech
Anti-HK2	ab209847	Abcam
anti-LDHA	19987-1-AP	Proteintech
anti-PKM2	#4053	Cell Signaling Technology
anti-MYC	#18583	Cell Signaling Technology
anti-β-catenin	#8480	Cell Signaling Technology
anti-MMP7	ab207299	Abcam
anti-MMP9	#13667	Cell Signaling Technology
anti-CCND1	#2978	Cell Signaling Technology
anti-METTL3	bs-17609R	BIOSS
anti-m6A	ab208577	Cell Signaling Technology
anti-Ki-67	ab15580	Abcam
anti-β-tubulin	bs-20694R	BIOSS
anti-β-actin	#4970	Cell Signaling Technology
anti-GAPDH	bs-0755R	BIOSS
anti-Lamin B	bs-24328R	BIOSS
