# Supplementary material for: N6-methyladenosine modification of REG1α facilitates colorectal cancer progression via β-catenin/MYC/LDHA axis mediated glycolytic reprogramming
Source: Cell Death Dis. 2023 Aug 25;14(8):557. doi: 10.1038/s41419-023-06067-6 (PMC10457312; doi:10.1038/s41419-023-06067-6)

Figure 3F

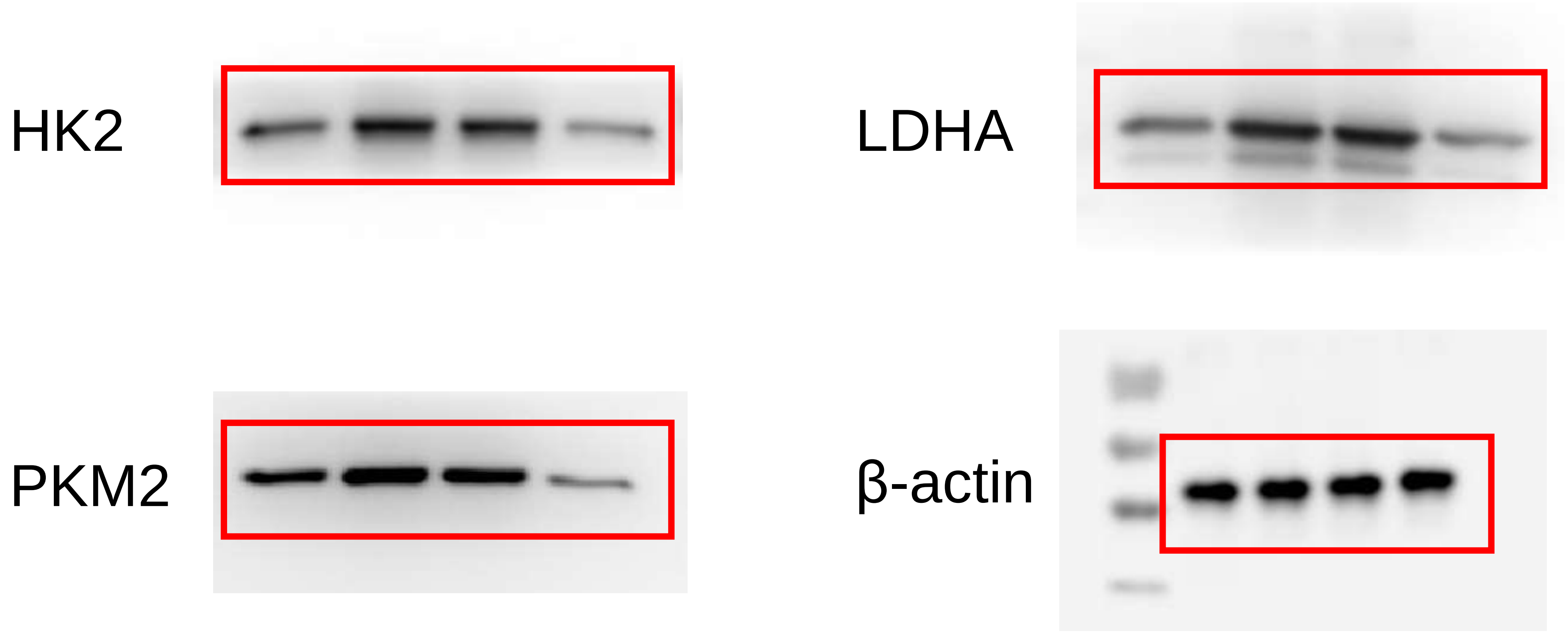

Figure 5C

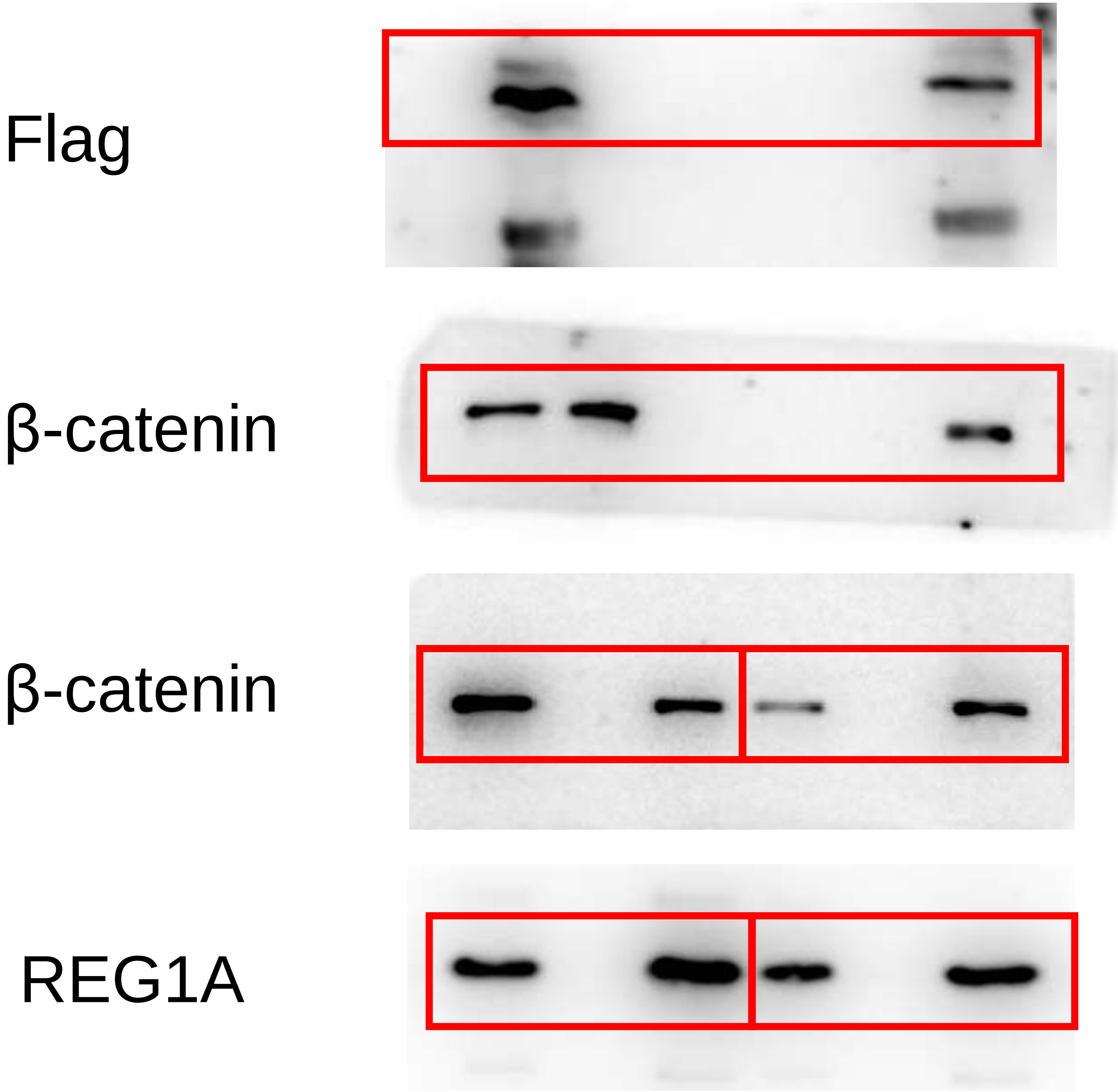

Figure 5K

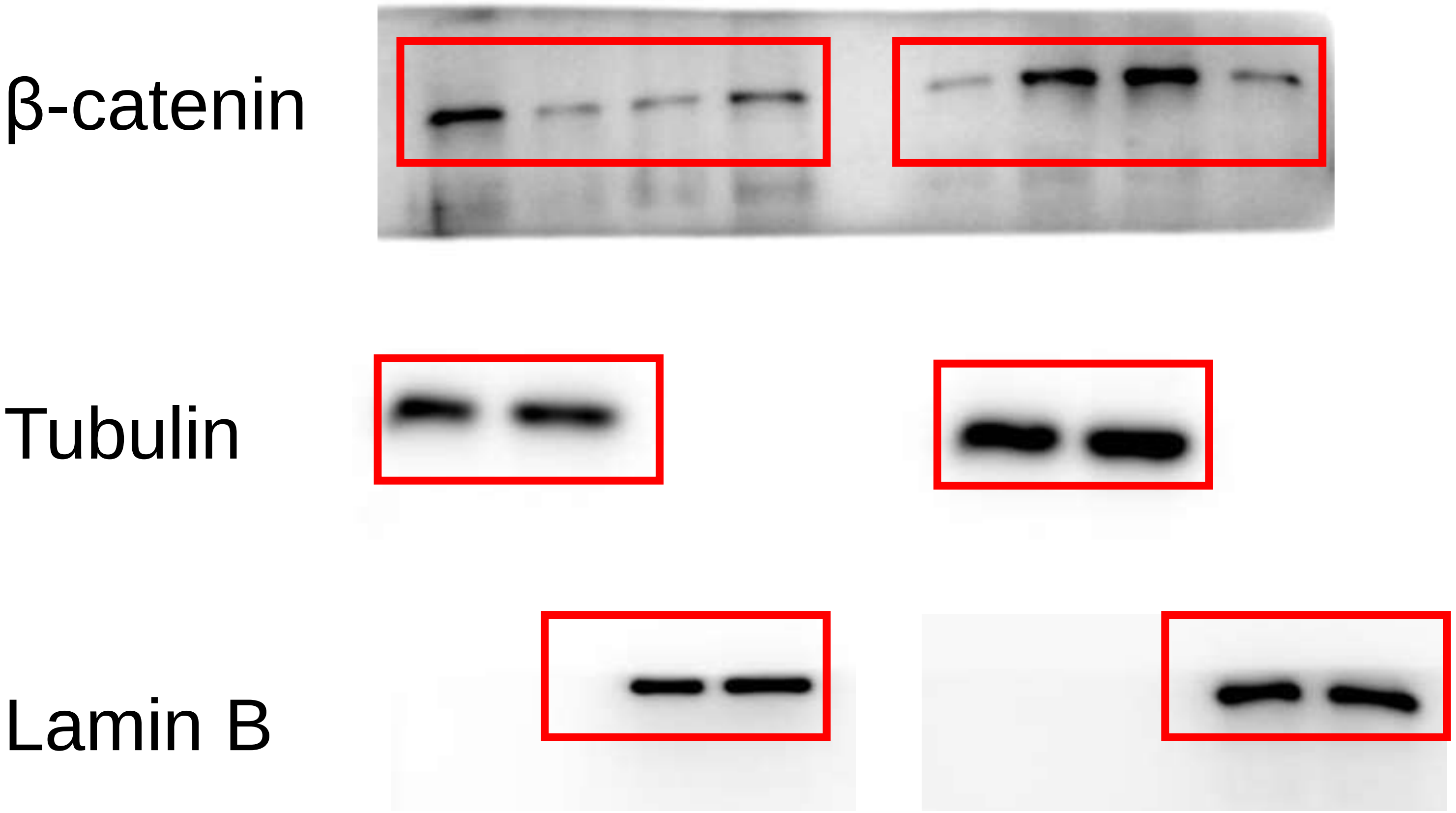

Figure 5L

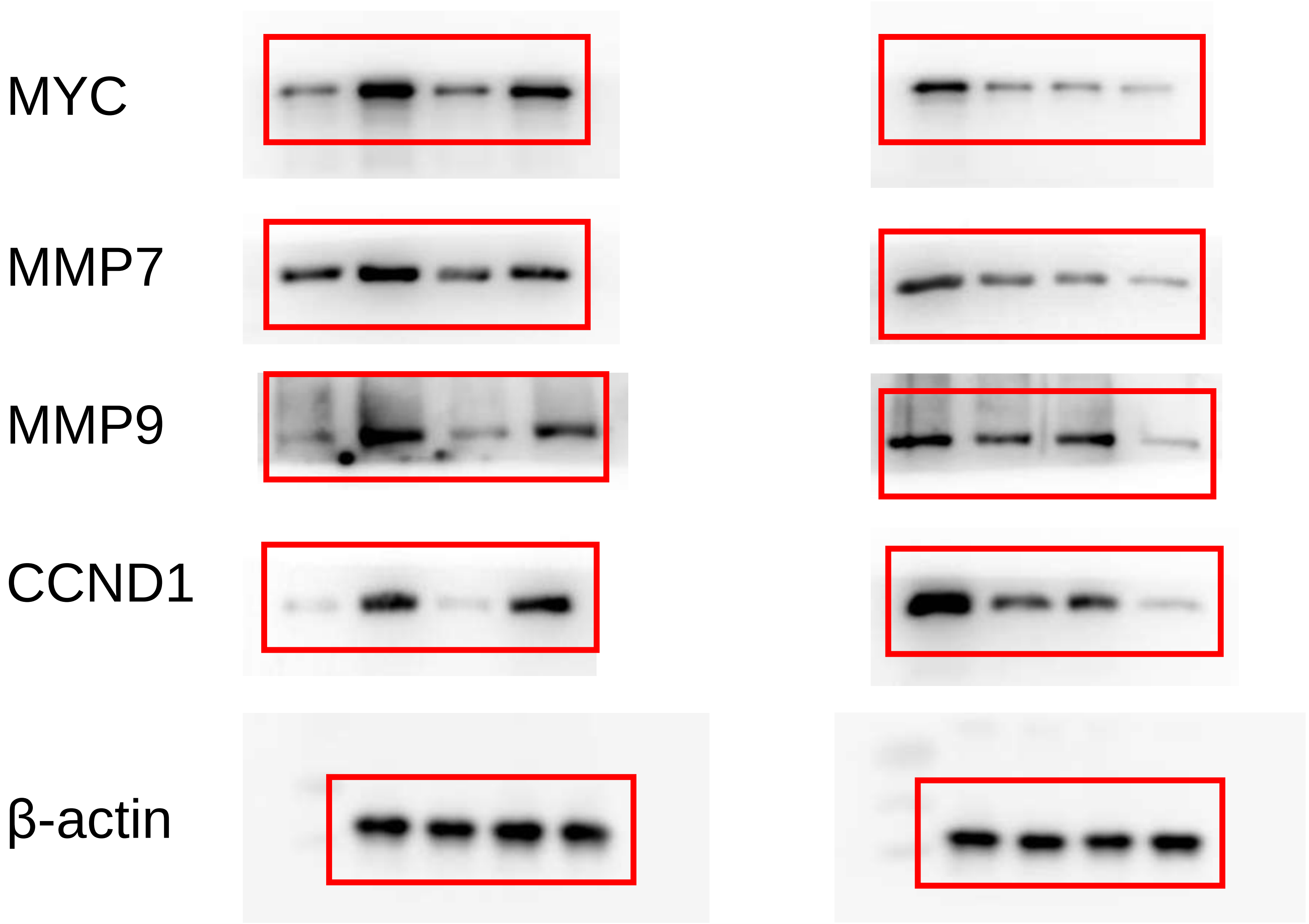

Figure 6D

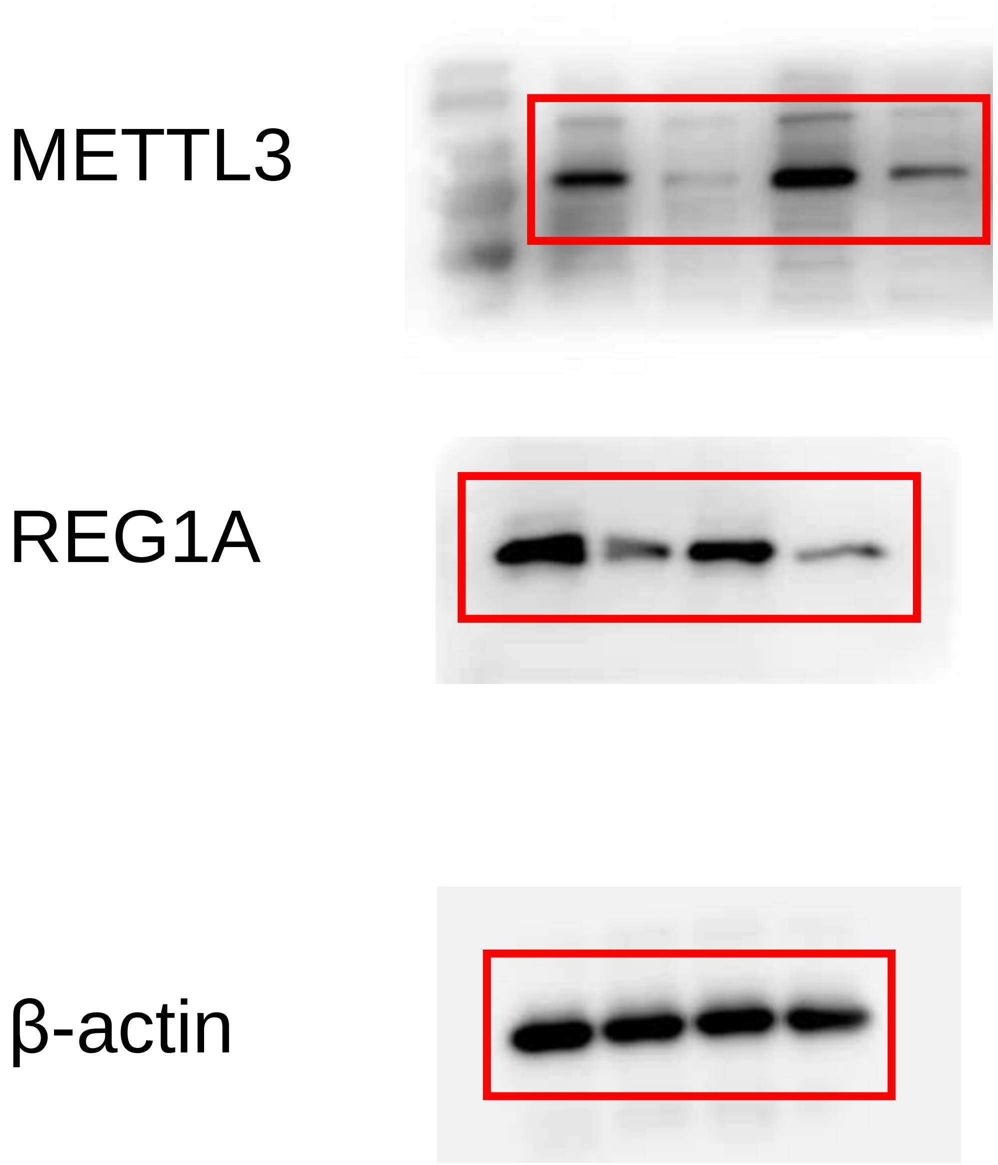

Figure 6E

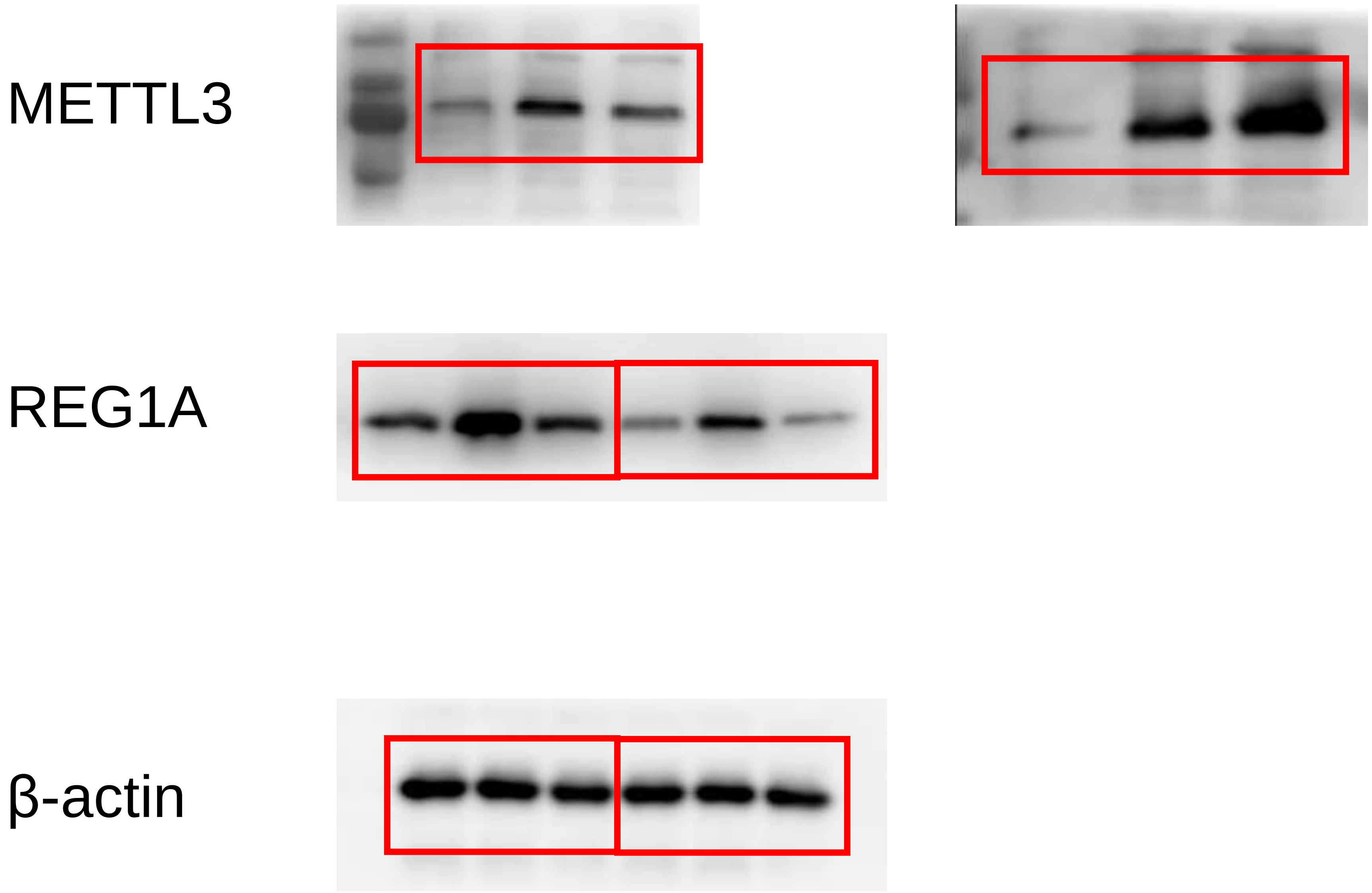

Supplement: Supplementary file 3 — Full and uncropped western blots [file 41419_2023_6067_MOESM3_ESM.pdf]
